# Harnessing Fermentation May Enhance the Performance of Biological Sulfate-Reducing Bioreactors

**DOI:** 10.1021/acs.est.3c04187

**Published:** 2024-02-01

**Authors:** Tomas Hessler, Susan T.L. Harrison, Jillian F. Banfield, Robert J. Huddy

**Affiliations:** † The Center for Bioprocess Engineering Research, University of Cape Town, Cape Town 7700, South Africa; ‡ Department of Chemical Engineering, University of Cape Town, Cape Town 7700, South Africa; § The Innovative Genomics Institute at the University of California, Berkeley, California CA94720, United States; ∥ The Department of Earth and Planetary Science, University of California, Berkeley, California CA94720, United States; ⊥ Environmental Genomics and Systems Biology Division, Lawrence Berkeley National Laboratory, Berkeley, California CA94720, United States; # The Future Water Institute, University of Cape Town, Cape Town 7700, South Africa; ∇ The Department of Environmental Science, Policy and Management, University of California, Berkeley, California CA94720, United States

**Keywords:** biological sulfate reduction, sulfate-reducing microorganisms, metagenomics, bioremediation, hydrogenase, microbial interactions

## Abstract

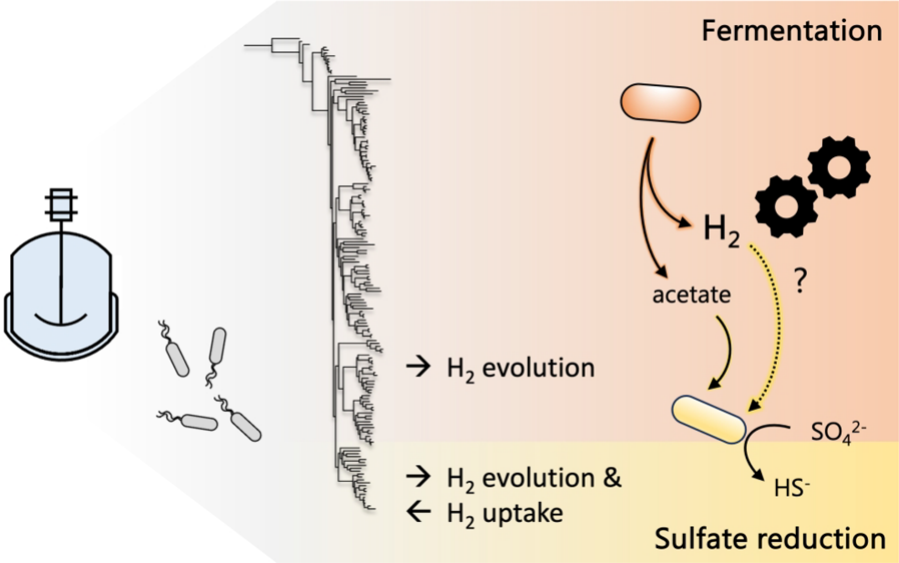

Biological sulfate reduction (BSR) represents a promising strategy for bioremediation of sulfate-rich waste streams, yet the impact of metabolic interactions on performance is largely unexplored. Here, genome-resolved metagenomics was used to characterize 17 microbial communities in reactors treating synthetic sulfate-contaminated solutions. Reactors were supplemented with lactate or acetate and a small amount of fermentable substrate. Of the 163 genomes representing all the abundant bacteria, 130 encode 321 NiFe and FeFe hydrogenases and all genomes of the 22 sulfate-reducing microorganisms (SRM) encode genes for H_2_ uptake. We observed lactate oxidation solely in the first packed bed reactor zone, with propionate and acetate oxidation in the middle and predominantly acetate oxidation in the effluent zone. The energetics of these reactions are very different, yet sulfate reduction kinetics were unaffected by the type of electron donor available. We hypothesize that the comparable rates, despite the typically slow growth of SRM on acetate, are a result of the consumption of H_2_ generated by fermentation. This is supported by the sustained performance of a predominantly acetate-supplemented stirred tank reactor dominated by diverse fermentative bacteria encoding FeFe hydrogenase genes and SRM capable of acetate and hydrogen consumption and CO_2_ assimilation. Thus, addition of fermentable substrates to stimulate syntrophic relationships may improve the performance of BSR reactors supplemented with inexpensive acetate.

## Abstract 2

This study offers strategies to improve the performance of bioreactors used for the remediation of sulfate-rich waste streams, such as acid rock drainage, enhancing the efficiency and cost effectiveness of this bioremediation process.

## Introduction

Sulfate-contaminated wastewater is generated by a number of industries, but posing the greatest environmental threat is acid rock drainage (ARD) arising from both current and historic mining activities. Abiotic and microbial oxidation of sulfidic ore produces low pH solutions with elevated sulfate and heavy metal concentrations.^1^ Although physical and chemical treatments are effective at remediating major ARD sites (e.g., mine water discharged from active mine workings), these processes are not suitable for the treatment of very common localized low-flow ARD because they demand substantial infrastructure and have high operating costs.

Decades of study have shown biological sulfate reduction (BSR) to be a promising low-cost approach for the bioremediation of diffuse sources of lower volume and less acidic ARD.^2,3^ BSR is catalyzed by a diverse group of anaerobic microorganisms known as sulfate-reducing microorganisms (SRM). Sulfate present in the ARD is used by the SRM within bioreactors as a terminal electron acceptor coupled to the oxidation of a supplied electron donor, resulting in the generation of sulfide and bicarbonate.^4^ The generated sulfide can be used to precipitate heavy metals in solution or be partially oxidized to elemental sulfur,^5^ a value-added product. Additionally, bicarbonate produced during this process can aid in neutralization of the solution. The choice of the supplied electron donor largely dictates the cost of an ARD-remediating BSR process.^6^

Many SRM readily consume hydrogen as an electron donor (eq 6) and use CO_2_ and/or acetate as a carbon source.^7,8^ The Paques Sulfateq treatment process, established as a large-scale treatment process for high-sulfate and heavy-metal-containing industrial effluents,^9^ uses hydrogen as the electron donor in gas lift reactors. However, at lower sulfate loadings, the electron donor is changed from hydrogen to ethanol or butanol to remain economically viable.

Lactate is often studied at a lab and pilot scale due to the favorable kinetics (eq 3 and 4, Table 1) and that the operating conditions for this electron donor have been optimized to favor the growth of SRM over fermentative microorganisms.^10^ A major limitation of BSR processes operated with various volatile fatty acids and complex carbon sources is the frequent buildup of acetate in the effluent of these reactors, even in the presence of high sulfate concentrations.^11,12^ This prevents safe discharge of these effluents due to their high organic content. Acetate oxidation is also found to be the rate-limiting step in these processes.^6^ The difficulty in establishing an active and robust acetate-utilizing SRM community in these reactor systems has puzzled researchers for some time.^13^ Acetate is of particular interest as an electron donor for sustainable ARD-remediating BSR processes (eq 1) due to its low cost compared to other electron donors and its potential to be sourced from various waste streams. Therefore, understanding the factors that enhance acetate-utilizing SRM’s growth within BSR reactors is important for the success of acetate-supplemented BSR systems as well as the efficient utilization of other electron donors that result in acetate production.

**Table 1:** Common Sulfate-Reducing and Fermentative Reactions, Adapted from Thauer et al.^8^

reaction equation	ΔG°′ (kJ/reaction)	
acetate^–^ + SO_4_^2–^ → 2 HCO_3_^–^ + HS^–^	–47.6	eq 1
propionate^–^ + 0.75 SO_4_^2–^ → acetate^–^ + HCO_3_^–^ + 0.75 HS^–^ + 0.25 H^+^	–37.7	eq 2
lactate^–^ + 0.5 SO_4_^2–^ → acetate^–^ + HCO_3_^–^ + 0.5 HS^–^	–80.2	eq 3
2 lactate^–^ + 3 SO_4_^2–^ → 6 HCO_3_^–^ + 3 HS^–^ + H^+^	–225.3	eq 4
3 lactate^–^ → acetate^–^ + 2 propionate^–^ + HCO_3_^–^ + H^+^	–70.0	eq 5
4 H_2_ + SO_4_^2–^ + H^+^ → HS^–^ + 4 H_2_O	–151.9	eq 6

Despite its promise, few ARD-remediating BSR bioreactors have been implemented and operated at scale, with the exception of passive systems with extended hydraulic retention time.^14^ The successful operation of BSR reactors faces several challenges, including the efficient utilization of the supplied electron donor due to competition between the SRM and other microorganisms within the microbial community. Further, achieving high reaction rates by overcoming the relatively low growth rates of SRM has been difficult.^15^ These challenges are intimately linked to the microbial community dynamics and the metabolic potential of the organisms in these systems, both of which remain little studied.

SRM have been extensively investigated in natural environments^16,17^ as these microorganisms play important roles in biogeochemical cycling. Pure and mixed SRM cultures have undergone thorough kinetic growth studies^10,18−20^ that have enabled the development of mathematical models used to optimize BSR processes.^21^ Complementing these culture-based studies, marker gene (e.g., the 16S rRNA gene) tracking has been used to uncover the factors that impact the microbial community composition within BSR reactor systems.^22−24^ However, genome-resolved metagenomic surveys of subsurface sediments and aquifers have revealed that SRM are more diverse than previously thought.^25^ This raises questions around the suitability of using only 16S rRNA gene surveys for the identification of SRM within bioreactor communities.

SRM in the environment are known to engage in syntrophic growth with many groups of microorganisms. In particular, SRM are often found with hydrogen-producing organisms such as fermentative bacteria, archaea, and green-sulfur bacteria.^26−29^ These hydrogen-generating microorganisms rely on other anaerobic microorganisms such as SRM to maintain low hydrogen partial pressures to allow fermentation to remain energetically favorable.^30,31^ These interactions are often discussed in environmental studies and in the case of methanogenic bioreactors.^32^ However, the relationship between fermentative organisms and SRM in BSR reactors is rarely discussed, and the potential for leveraging this synergistic relationship to improve reactor performance is yet to be proposed.

Many SRM are known to consume molecular hydrogen as an energy source, but some are also able to produce hydrogen in the absence of sulfate when growing fermentatively.^33,34^ FeFe hydrogenases are commonly used by fermentative bacteria to recycle electron carriers, which become reduced during fermentation, leading to hydrogen generation.^35^ These hydrogenases are most associated with hydrogen production, while many groups of 1 NiFe hydrogenases are associated with hydrogen uptake.^36^

Here, we report the genome-resolved metagenomic survey of microbial communities of six simultaneously inoculated continuously operated BSR reactors. By employing multiple bioreactors, we aimed to study many stable microbial communities, arising from a single inoculum, that facilitate effective sulfate-reducing reactor performance. Some of the bioreactor performance data were previously published,^37−39^ including the performance of the packed bed reactors and the acetate channel reactor, but in the current study, we integrate performance data from all six bioreactors to contextualize the metagenomic results. Prior to this study, there was essentially no genome-resolved metagenome information available regarding the biological underpinnings of the biological sulfate reduction bioreactors. We were motivated to analyze the microbial communities in the bioreactors using genome-resolved metagenomics to (a) evaluate the microbial community complexity, (b) determine which community members likely perform the critical process steps, (d) identify potential synergistic effects arising from organism–organism interactions, and (e) develop the basis for hypotheses that could be tested in a future work.

**Figure 1:**
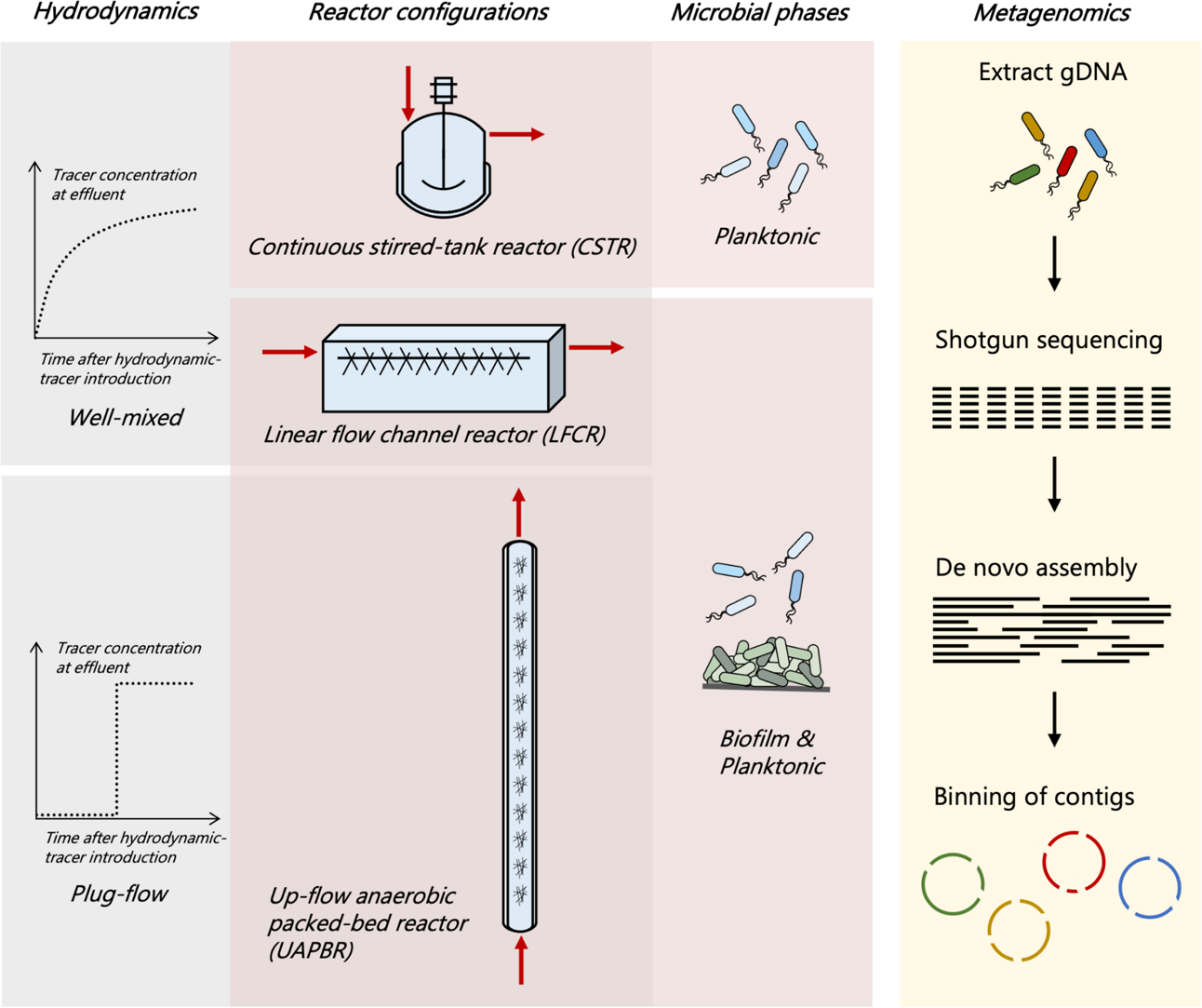
Schematic diagrams of the bioreactors used in this study, highlighting their differences in the prevailing hydrodynamics and the phases of microbial growth that they support. A panel illustrating the rationale for genome-resolved metagenomics used to study the microbial communities within these bioreactors is shown on the right.

Although bioremediation of ARD can be performed successfully in a single reactor,^40^ the bioreactors used in this study were intended to follow neutralization and heavy metal removal steps in the ARD remediation process and were, therefore, operated with neutral, defined drainage. We monitored the performance of these reactors as we iteratively reduced the operated hydraulic retention times from 4 to 1 day. Genome-resolved metagenomics was used to characterize the communities only at a four-day hydraulic retention time (HRT), after which they were monitored using 16S rRNA gene amplicon sequencing. We employed three reactor types, namely, up-flow anaerobic packed bed reactors (shortened to “packed bed reactor”, UAPBR), linear flow channel reactors (shortened to “channel reactor”, LFCR), and continuous stirred tank reactors (shortened to “stirred tank reactor”, CSTR; Figure 1). Packed bed reactors are plug-flow-governed configurations commonly used in BSR research.^2^ These reactors enable high biomass retention in the form of biofilms that colonize the packing material. Plug-flow results in the stratification of the type and rate of the reactions along the reactor length. The channel reactor is a newly developed reactor configuration that achieves complete mixing passively within a single HRT.^41^ Carbon microfibers were incorporated into this configuration to support biofilm formation. In contrast, stirred tank reactors are well-mixed reactors with low surface area/volume ratios that result in predominantly planktonic microbial communities. Within this study, the stirred tank reactors allow the investigation of planktonic microbial communities in the absence of biofilm communities and thereby rapid community dynamics in response to operating conditions, governed dominantly by biokinetics. Two of each reactor type were operated, one supplemented with (1.2 g/L) sodium lactate and the other with (0.92 g/L) sodium acetate as the primary electron donors. We also included lower concentrations of (0.23 g/L) citrate and (0.4 g/L) yeast extract in the media intended to prevent salt precipitation and as a nutrient source, respectively. Serendipitously, the inclusion of citrate and yeast extract appears to have promoted the growth of fermentative microorganisms, which are likely contributing to hydrogen production in our bioreactors.

The distinct selective pressures, which resulted from the differences in reactor hydrodynamics, supplied electron donors, and their capacity to support microbial biofilms was intended to lead to the divergence of the composition of these microbial communities from each other and the original inoculum, generating many valuable microbial communities suitable for studying BSR using metagenomics.

## Results and Discussion

### Six Reactors’ Performance

**Figure 2:**
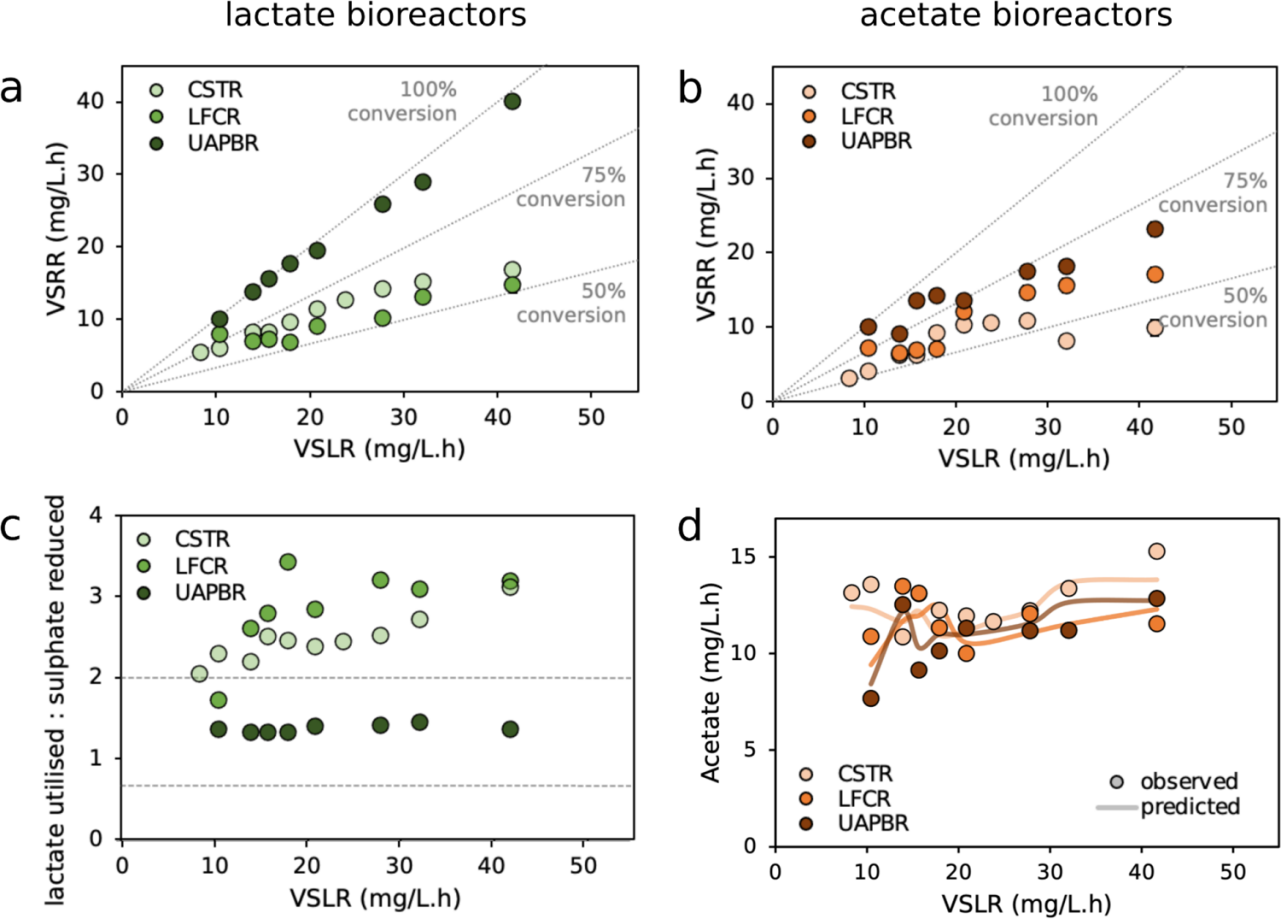
Volumetric sulfate reduction rates achieved by the (a) acetate- and (b) lactate-supplement bioreactors at increasing volumetric sulfate loading rates (increased through the reduction of the applied hydraulic residence time). These reactors were CSTRs, LFCRs, and UAPBRs. (c) The efficiency of lactate oxidation was assessed by considering the ratio of lactate utilized to sulfate reduced. The theoretical lactate utilized to sulfate reduced ratios corresponding to incomplete lactate oxidation (eq 5, 2:1) and complete lactate oxidation (eq 4, 2:3) by SRM is shown as dotted lines. (d) The acetate concentrations in the acetate-supplemented bioreactors were measured and independently predicted based on the observed sulfate reduction coupled to acetate oxidation (eq 1).

We monitored the sulfate reduction performance of the six bioreactors during chemically defined steady states at iteratively reduced hydraulic retention times. The performance of the reactors was evaluated primarily on the volumetric sulfate reduction rates (VSRR) and sulfate conversions these reactors achieved during the HRT study (Figure 2a,b). The volatile fatty acid consumption and accumulation in the reactors were monitored by HPLC and stoichiometrically coupled to the sulfate-reducing performance of the reactors according to reactions shown in Table 1 (Figure 2c,d).

The stirred tank reactors showed some of the poorest sulfate-reducing performance, as had been anticipated based on the low biomass retention of these configurations. However, both maintained sulfate-reducing performance at the shortest applied HRT, indicating that all SRM present in the reactors were able to maintain a growth rate equal to or greater than the applied dilution rate of 0.042 h^–1^. The elevated performance of the acetate-supplemented channel reactor, compared to the stirred tank reactor, was previously attributed to the immobilization of cells within biofilms.^38^ The promotion of biofilms within the channel reactor enabled a 10-fold higher biomass retention than the stirred tank reactor (data 6, 11, and 12).

Of the three reactor configurations, the two packed bed reactors exhibited the greatest VSRR throughout the HRT study. The principal difference between the packed bed reactors and the other configurations is their plug-flow-governed hydrodynamics (Figure 1), which gave rise to gradients of reactants and products throughout the reactors (Supplementary Data 13, 14, and 17). The predominant electron donor used for sulfate reduction in the lactate-supplemented packed bed reactor changed from lactate in the first third, to predominantly propionate (with some acetate) in the second, and predominantly acetate in the final third of the reactor. The efficient use of the products of lactate oxidation by fermenters (propionate and acetate, eq 5) and SRM (acetate, eq 3) enabled the packed bed reactor to achieve high sulfate conversions for the duration of the study. This is illustrated in Figure 2C where the packed bed reactor was the most efficient system for oxidation of lactate, due to the subsequent oxidation of propionate and acetate in this system, reiterating our previous claims^37^ that plug-flow systems allow better performance of BSR reactor systems.

The concentration of acetate in acetate-supplemented systems was monitored and independently predicted based on the observed sulfate reduction and putatively linked stoichiometrically to acetate oxidation according to eq 1. We also accounted for acetate generated through the oxidation of yeast extract, which was previously quantified by varying the yeast extract concentration and monitoring change in acetate concentrations.^38^ The average difference between the observed and predicted acetate concentrations leaving the stirred tank, channel, and packed bed reactors was just 6, 9, and 6%, respectively (Figure 2D). The agreement between the predicted and observed acetate concentrations provides evidence that acetate removal and sulfate reduction are linked.

### Kinetic Modeling of the Packed Bed Reactors’ Performance

**Figure 3:**
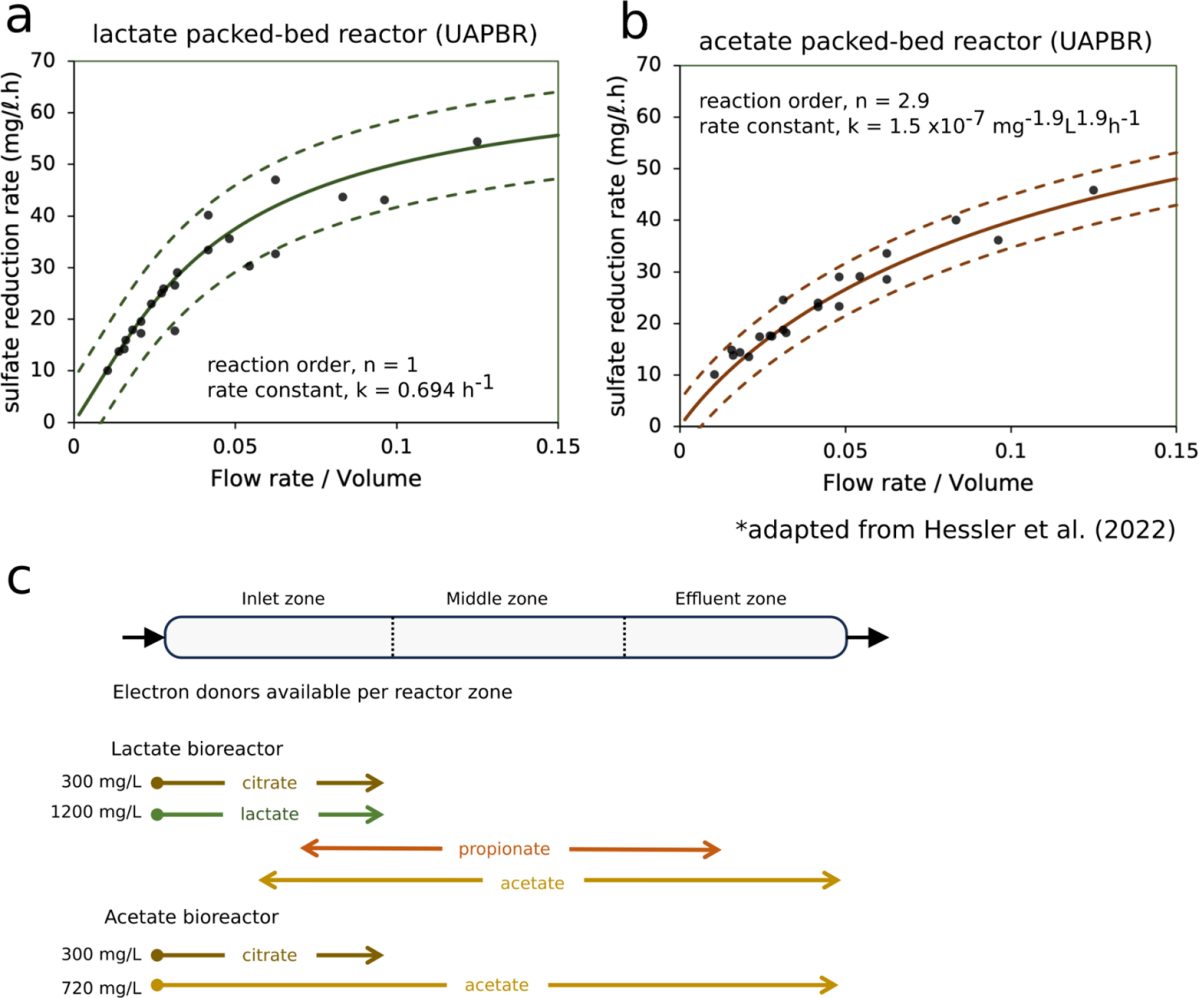
Observed and modeled sulfate reduction rates observed in the (a) lactate- and (b) acetate-supplemented UAPBRs at a range of flow rate-to-volume ratios. Adapted from Hessler et al.^39^ The determined reaction order (*n*) and rate constant (*k*) are shown. (a, b) The modeled reaction rates are shown over a range of applied dilution rates where the starting substrate concentration (*C*_0_) is 1,000 mg/L with observed reaction rate data from the inlet zones (0.33 L), composite inlet and middle zones (0.66 L), and entire reactors (1.0 L). (c) The availability of different organic electron donors in the UAPBRs, quantified by HPLC, is shown schematically.

We previously modeled the rate of sulfate reduction observed in the packed bed reactors as irreversible n^th^-order reactions (eq 7) along an ideal plug-flow reactor (eq 8) according to the derived eq 9 and eq 10 (Methodology).^39^ This analysis aimed to determine the reaction order and rate constant across the different packed bed reactors in a generalized sulfate reduction model assuming a constant rate constant. We anticipated the rate of sulfate reduction in the lactate-supplemented packed bed to substantially decrease once lactate was depleted, leading to a higher modeled reaction order. Lactate was rarely detected beyond the first third of the reactor. This required the SRM in the remainder of the column to consume the less energy-rich propionate and acetate. Surprisingly, the volumetric sulfate reduction rates in the lactate-supplemented packed bed reactor were best described as a first-order reaction, indicating that the modeled sulfate reduction rate was consistently proportional to the sulfate concentration but unaffected by changing electron donors^39^ (Figure 3a). This is noteworthy because the oxidation of propionate and acetate coupled to sulfate reduction is less energetically favorable than that linked to lactate oxidation (Table 1). As the energetics of these reactions are very different, yet sulfate reduction kinetics were unaffected by the type of electron donor available, we hypothesize that an alternative electron donor was being oxidized by the SRM that we had not considered. The cell density within the biofilms throughout this reactor also decreases substantially between the inlet and effluent (Supplementary Data 18 and 19), meaning that the specific sulfate reduction rate of lactate oxidizing SRM is lower than that of the propionate and acetate-oxidizing SRM. A potential explanation for this phenomenon is that SRM were also consuming hydrogen (eq 6), a highly favorable reaction, produced through fermentation in the reactor. We had expected the acetate-packed bed reactor to exhibit a near first-order volumetric sulfate reduction rate, given the very high affinity of SRM for acetate^19^ and, therefore, the concentration of acetate having little effect on the growth rate of these SRM. However, we find that the volumetric sulfate reduction rates demonstrated by this reactor were best modeled with a reaction order of 2.9 (Figure 2b), indicating that a substantial decrease in growth rate is observed in the reactor despite consistently available acetate. This strongly suggests that the oxidation of additional electron donors, apart from acetate, was occurring in this reactor.

### Genome Recovery

The 34 metagenomes (17 samples in duplicate), representing the initial inoculum and reactor microbial communities at a four-day HRT, were individually assembled, and 163 draft microbial genomes were reconstructed. Based on the inventory of bacterial and archaeal single-copy genes, the average estimated completeness of the recovered genomes was 95%, with 127 genomes having an estimated completeness of >90.0% and <5.0% contamination (Supplementary Data 20–23). At least 30 draft genomes were reconstructed from each sample, and the genomes represent all of the abundant organisms in each reactor. The average and minimum proportion of the reads from each metagenome that mapped to binned contigs (≥1000 bp) were 89 and 85%, respectively. Thus, the genomes well represent the organisms present in the bioreactors. The bioreactor communities have relatively low species diversity, with an average Shannon index of 3.0. This was expected for each of the bioreactors due to the lower metabolic diversity they select for together with the controlled conditions and steady-state conditions that result.^42^ However, many of the genomes were found for organisms that were dominant in only a subset of the samples. We attribute the large number of high-quality recovered genomes to the heterogeneity under the physiochemical conditions generated across the varying bioreactor environments.

The reactors were supplemented with the methanogenic inhibitor BESA at the time of inoculation to provide an initial advantage to acetate-oxidizing SRM over any methanogen for available acetate. A single archaeal genome (for a Methanomassiliicoccales) was recovered but was present only in the inoculum. Genes for methanogenesis were not detected in any of the remaining genomes (Supporting Information, data 25). The remaining 162 bacterial genomes were classified to 16 phyla but were predominantly assigned to Alphaproteobacteria (14 genomes), Betaproteobacteria (6), Myxococcota (Deltaproteobacteria; 22), Gammaproteobacteria (12), Campylobacterota (Epsilonbacteria; 5), Bacillota (29), Bacteroidota (28), Synergistota (15), and Spirochaetota (12).

### Prevailing Metabolisms

**Figure 4:**
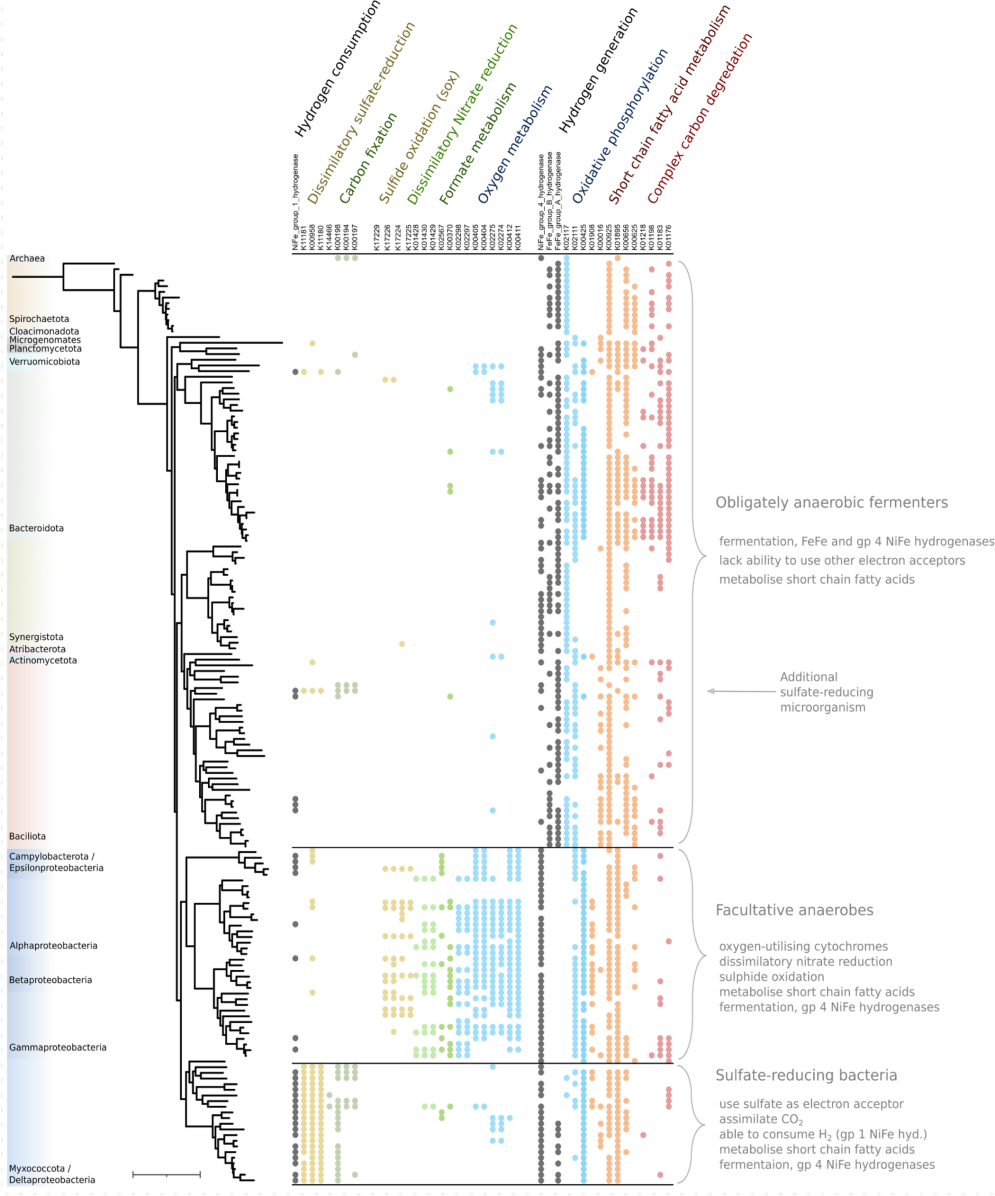
Phylogenetic tree based on 16 concatenated ribosomal proteins (left) and the presence and absence of metabolic genes encoded by the corresponding 163 recovered microbial genomes (right). The major metabolic strategies enabled by these encoded genes are annotated on the right, showing that most of the organisms fall into one of three metabolic strategies that align closely with their microbial phyla. These data can be found in Supplementary Data 26.

**Figure 5:**
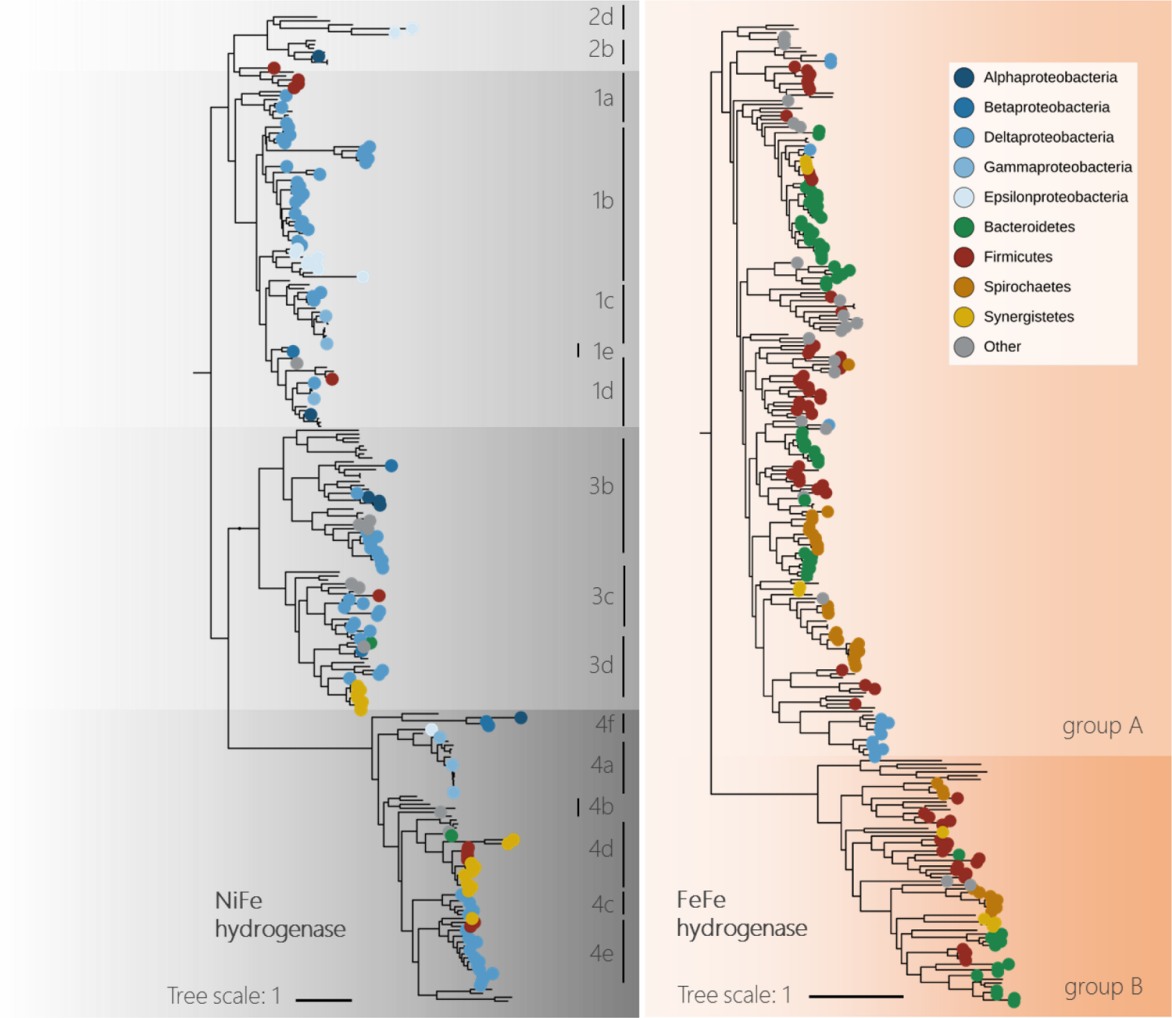
Phylogenies and classification of the 321 NiFe and FeFe hydrogenase protein sequences identified from 130 of the total 163 genomes recovered from the bioreactors of this study. Reference sequences are shown without phylum annotation. Hydrogenase genes were identified using custom-built HMMs and were further classified according to HydDB.^80^ FeFe group A subgroups cannot be resolved phylogenetically but were further classified based on the immediately downstream catalytic subunit (Supplementary Data 27).

Evaluation of the metabolic genes encoded in the 163 genomes revealed three predominant metabolic strategies: sulfate reduction coupled to volatile fatty acid and hydrogen oxidation, strict anaerobes reliant solely on fermentation, and last a metabolically versatile group of Pseudonomata capable of sulfide oxidation linked with aerobic respiration or dissimilatory nitrate reduction and also capable of fermentation of volatile fatty acids in the absence of oxygen or nitrate (Figure 4). These three prevailing forms of encoded metabolisms are largely distributed separately across phylogeny, with genomes within phyla showing a large degree of metabolic redundancy. Hydrogen metabolism was widespread across the recovered genomes, with a total of 321 hydrogenases encoded in 130 genomes (Figure 5). Unfortunately, we did not assay for hydrogen in our bioreactors and, therefore, all further evidence of hydrogen exchange is sourced from metabolism encoded by the recovered genomes.

The genomes encoding strictly anaerobic, fermentative metabolisms commonly encode group 4 NiFe and groups A and B FeFe hydrogenases together with oxidative phosphorylation pathways, including V-type ATPases. Many Bacteroidota in this group also encoded genes for complex carbon degradation, while the other organisms, mainly Bacillota, Synergistota, and Spirochaetota, are less metabolically versatile and are restricted to the fermentation of simpler organic compounds. Some Bacteroidota^43^ and Spirochaeta^27^ have been shown to perform anaerobic dead cell scavenging. These organisms have been shown to produce hydrogen during anaerobic scavenging and require a syntrophic partner (e.g., methanogens or SRM) to consume the produced hydrogen. It is possible that Bacteroidota, encoding an array of complex carbon degrading enzymes, may have a similar role in our bioreactors.

A total of 182 FeFe hydrogenases were identified in the genomes of Bacteroidota, Spirochaetota, and Bacillota alone (Figure 5). FeFe hydrogenases are well-studied and most commonly associated with H_2_ evolution.^35,44^ The most commonly encoded were the trimeric group FeFe group A3 hydrogenase, which reversibly bifurcates electrons between H_2_, and ferredoxin and NAD.^45^ Genes encoding Rnf complexes were also common in these genomes. Rnf complexes are involved in electron transfer indirectly from the oxidation of organic compounds used for the translocation of ions across the cytoplasmic membrane to create of a proton motive force.^46^ Pyruvate ferredoxin oxidoreductases (porA) implicated in hydrogen production were also present in some Bacillota genomes. Hydrogen can be generated by formate oxidation catalyzed by a NiFe hydrogenase^47^ or group A4 FeFe hydrogenases^45^ coupled to a formate dehydrogenase (*Fdh*); however, formate dehydrogenases were only typically found in low-abundance genomes.

Hydrogen-evolving group 4 NiFe hydrogenases were commonly found in Synergistota genomes (Figure 5). For example, group 4d NiFe hydrogenases, in which hydrogen evolution is linked to ferredoxin oxidation and sodium translocation,^48^ were identified in 12 of the 15 recovered Synergistota genomes (Figure 5). These genomes commonly encoded additional FeFe hydrogenases and bidirectional NADH-linked NiFe group 3d hydrogenases (Figure 5). Each of these genomes encoded a number of genes relating to amino acid catabolism, acetogenesis and genes encoding oxaloacetate decarboxylases necessary for anaerobic citrate consumption,^49^ and other processes. However, none encoded the methylmalonyl-CoA or acrylyl-CoA pathway nor any lactate dehydrogenases needed for lactate oxidation. One of these microorganisms, genome Synergistales_64_17, was dominant across most of the bioreactor communities, particularly the acetate- and lactate-supplemented stirred tank reactors. Pyruvate ferredoxin oxidoreductases (*porA*) implicated in hydrogen production were also common in Synergistota genomes. Thus, we infer that Synergistota bacteria are important contributors to hydrogen production in the bioreactors, likely coupled to the fermentation of amino acids and possibly citrate.

A large proportion of genomes classified as Baciliota encoded lactate dehydrogenases (K00016, Figure 4). A *Veillonella-*classified genome, a genus commonly implicated in lactate oxidation in other environments,^50^ was found in high abundance in the lactate-stirred tank and the inlet zone of the lactate-packed bed reactor. Its genome encodes a lactate dehydrogenase together with the methylmalonyl pathway for the oxidation of lactate with propionate as a byproduct. The capacity to consume lactate was rare outside of Baciliota and SRM, and therefore, we attribute the majority of the fermentative oxidation of lactate and in our systems to these Baciliota.

The facultative aerobes, all of which are Pseudomonata, encoded oxidative phosphorylation electron transport chains including cbb3 oxygen reductases^51^ and bd-quinol oxidases,^52^ which have both been characterized with a high affinity for oxygen. However, functionally, the heme-copper cbb3 oxygen reductase would be inhibited by the hydrogen sulfide^53^ present in the reactors at up to 0.33 g/L. Thus, aerobic metabolism would be reliant on sulfide-resistant bd-quinol oxidases^54^ in these reactors. This functionality is important to note because influent solutions in real-world BSR applications contain trace oxygen, and its removal is beneficial to reactor performance. Some of these Pseudomonadota also encoded sulfide (*sox*) gene pathways, indicating that trace oxygen consumption could be linked to the oxidation of the sulfide produced by SRM. Alphaproetobacteria and Gammaproteobacterial genomes showed some of the highest prevalence of genes required for branched-chain amino acid catabolism (Supplementary Data 29). These may have afforded these organisms a competitive advantage at metabolizing some the components of the yeast extract in the medium as electron donors. Dissimilatory nitrate reduction genes were also common in these organisms. No nitrate was included in the medium and although feasible that ammonia supplied in the medium may have been oxidized to nitrate, we were unable to identify genes related to anaerobic ammonia oxidizing (anammox) or nitrification in any of the recovered genomes. Low concentrations of sulfide, lower than observed in our bioreactors, are known to inhibit the growth of anammox^55,56^ and nitrifying bacteria^57^ and may account for their absence from our bioreactors. The most abundant facultative aerobe in the bioreactors was a *Paracoccus* (genome Paracoccus_versutus_68_139) that was found in the planktonic communities throughout the acetate and lactate-packed bed reactors. This genus is known to be important for nitrogen removal in wastewater processes and may well have been using low concentrations of nitrate linked to sulfide oxidation. However, the observed sulfide concentration in our bioreactors was largely in agreement with the stoichiometrically predicted sulfide concentration based on the observed sulfate reduction (Supplementary Data 14). These genomes also encode group 4 NiFe hydrogenases, indicating their capacity to perform fermentation, together with the oxidation of volatile fatty acids, in the absence of oxygen.

Homoacetogenesis appears to be absent in our bioreactors, based on the poor representation of complete Wood–Ljungdahl pathways in non-SRM genomes and the absence of common homoacetogenic taxa.^58^ The absence of alternative electron acceptors in our medium together with the lack of gene pathways in these metagenomes relating to other forms of chemolithotrophy further indicates that fermentation and respiration using sulfate and potentially trace oxygen are the only forms of metabolism that could be occurring in these reactor systems.

### Sulfate-Reducing Microorganisms

Twenty-two of the recovered genomes were for organisms predicted to be capable of dissimilatory sulfate reduction based on the recovery of genes encoding adenylylsulfate reductases, sulfate adenylyltransferases, and dissimilatory sulfite reductases (Figure 4). Twenty-one of these SRM were classified as Myxococcota (Deltaproteobacteria), and a single genome was classified to the Clostridial genus *Desulfomaculatum*. The 22 SRM collectively encoded for 77 hydrogenases, the majority of the 22 genomes encoding 1a or 1b NiFe hydrogenase (Figures 4 and 5), hydrogenases that are responsible for H_2_ uptake and are well-studied in SRM.^37^ Groups 3b and 3d were also common in the SRM genomes. These hydrogenases are responsible for electron bifurcation coupled to NADPH and bidirectional hydrogen activity coupled to NADH, respectively.^45,59,60^ Many *Desulfovibrio* genomes and the recovered *Desulfomicrobium baculum* genome encoded group A FeFe hydrogenases and many of these SRM encoded NiFe group 4e hydrogenases, bidirectional hydrogenases that couple ferredoxin oxidation to H^+^ reduction.^61^ This is indicative of these microorganisms’ capacity to contribute to H_2_ evolution. This dual metabolic capacity is known to allow SRM to transition from sulfidogenic to hydrogenotrophic metabolism in the absence of sulfate.^62^ Pyruvate ferredoxin oxidoreductases (*porA*), which catalyze the oxidation of pyruvate linked to the reduction of ferredoxin needed for hydrogen evolution, were also common in SRM.

**Figure 6:**
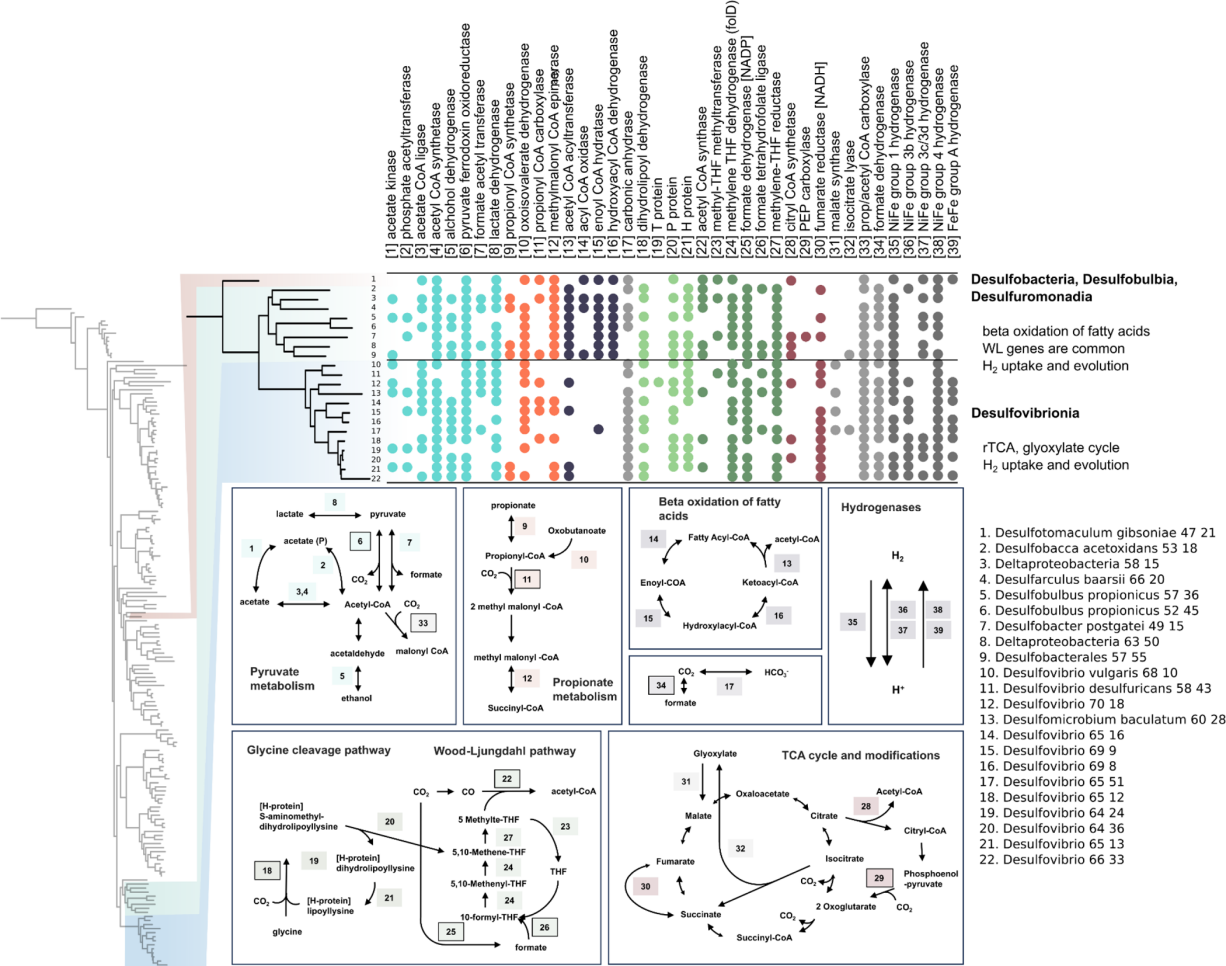
Phylogenetic tree presented in Figure 4 (left) focuses on microorganisms capable of dissimilatory sulfate reduction. A presence absence table (top) of specific metabolic genes, emphasizing distinctions between Desulfovbrionales and other classes of SRM. The numbers on the metabolic schematics (bottom) correspond to genes represented in the presence–absence table. Data represented in this plot can be found in Supplementary Data 28.

The recovered SRM exhibit a diverse array of metabolic enzymes that reflect their metabolic versatility (Figure 6), consistent with prior expectations.^4^ The majority of the SRM possessed a number of genes associated with acetate metabolism. It is important to note that many of these enzymes are bidirectional (Figure 6), meaning it is difficult to differentiate between acetate production and acetate assimilation. Within this group, we identified *Desulfovibrio* and *Desulfobacter* genomes that encoded propionyl-CoA synthetase, an enzyme that is needed for propionate metabolism (Figure 6). The beta oxidation of longer chain fatty acids was exclusively found in non-Desulfovibrionales SRM. Additionally, genes involved in carbon fixation strategies were identified, including the Wood–Ljungdahl pathway in *Desulobacter*, *Desulfobulbus*, and *Desulfomaticulum*, as well as enzymes of the reductive tricarboxylic acid (rTCA) cycle encoded in *Desulfovibrios*. Among the rTCA genes, fumarate reductase was notably prevalent in the *Desulfovibrio* genomes. These SRM likely use this enzyme to operate part of the tricarboxylic acid (TCA) cycle in reverse to generate essential intermediates without the loss of CO_2_ through the standard direction of the TCA cycle (Figure 6) when organic carbon sources are limiting. Similarly, several SRM employed either a partial or complete glyoxylate shunt to bypass specific steps of the TCA cycle, preventing this same loss of CO_2_ incurred during the typical TCA cycle.

Aside from the dedicated carbon fixation pathways, the SRM also encoded a number of carboxylases, which can be used for assimilation of CO_2_ into biomass, together with an almost ubiquitous distribution of carbonic anhydrases. It is important to note that complete carbon fixation pathways, such as the Wood–Ljungdahl and rTCA cycles, would not be essential for CO_2_ assimilation in our bioreactors as acetate was always present. Instead, SRM could also employ encoded propionyl-CoA carboxylases for the conversion of propionyl-CoA and CO_2_ to methylmalonyl-CoA, acetyl-CoA carboxylases for the conversion of acetyl-CoA and CO_2_ to malonyl-CoA, and phosphoenolpyruvate carboxylase for conversion of phophoenol-pyruvate and CO_2_ to oxoglutarate. This is in accordance with the SRM literature where members of multiple genera are able to assimilate CO_2_ together with acetate while performing sulfate reduction and hydrogen oxidation.^63−65^

### Bioreactor Community Composition

**Figure 7:**
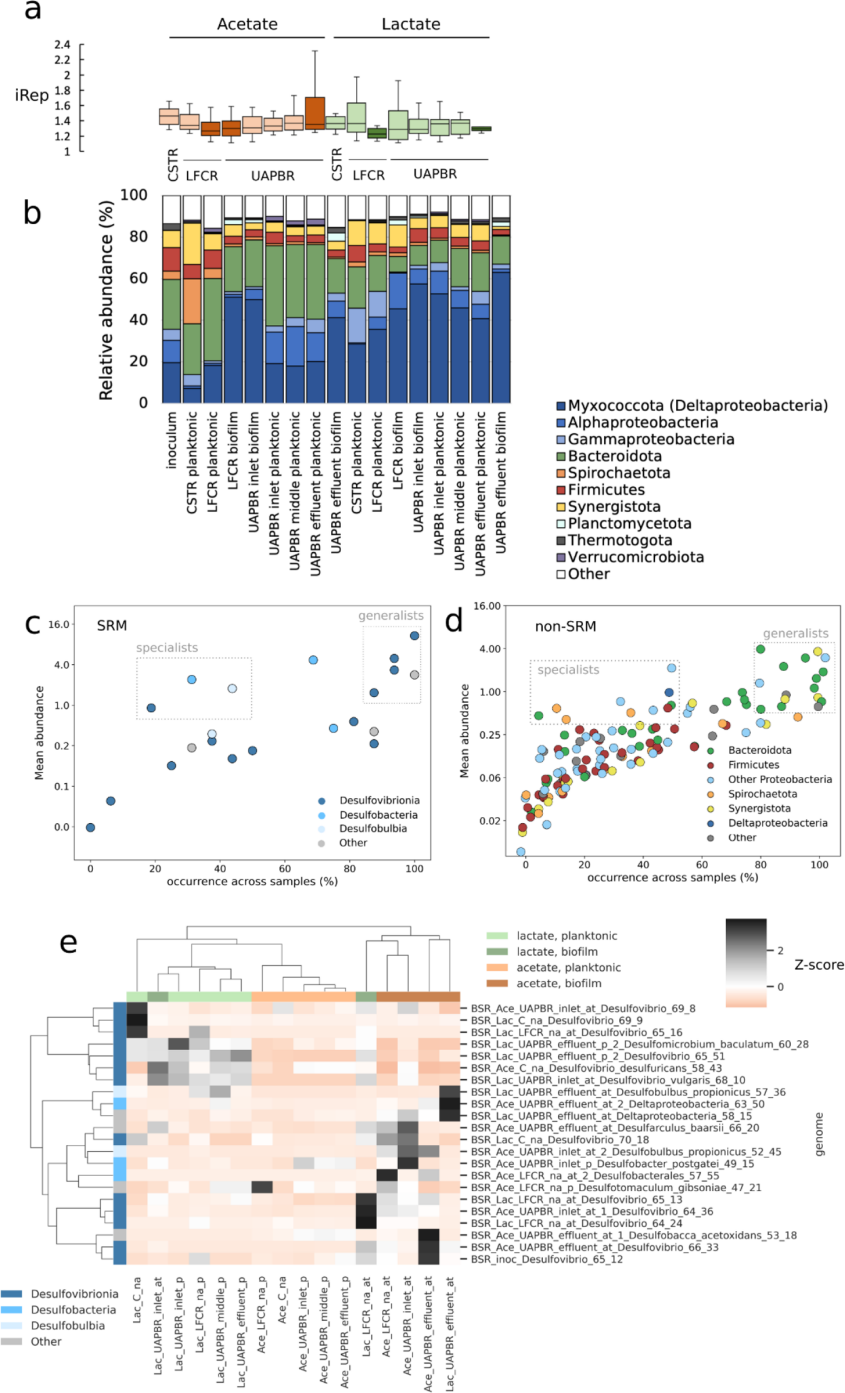
(a) iRep characterizing the microbial replication rates of microorganisms within the biofilm (dark) and planktonic (light color) bioreactor communities of the six bioreactors at a four-day HRT steady. (b) The composition of these microbial communities is shown at the phyla level. The organisms, (c) SRM and non-SRM and (d) which make up these communities, are shown by their mean abundance across all reactor samples and the proportion of total samples in which they were detected. (e) A hierarchically clustered heatmap of *z* scores is shown to indicate the distribution of SRM across the different bioreactor communities. These scores represent the direction and number of standard deviations from the mean relative abundance of an organism across all communities.

Indices of replication^66^ (iRep) were determined to assess microbial replication rates across the different reactor environments (Figure 7a). Most of these reactor communities showed narrow and similar distributions in represented iRep values. The narrow ranges indicate that the microbial communities were stable at the time of sampling. Where biofilms were present, the median iRep values for bacteria in biofilms were generally lower than those of bacteria in the planktonic communities that occupied the same reactor zones, although the differences were not statistically significant. The generally comparable replication rates for planktonic and biofilm-associated bacteria is of particular interest as biomass quantification found biofilms contribute up to 100-fold more cells per reactor volume than planktonic communities within the same reactor zone (Supplementary Data 6, 11, 12, 18, and 19). The relative abundance of the SRM in all zones was, on average, twice that in biofilms compared with planktonic communities. The contribution of these biofilms to biomass retention within the reactors, the representation of SRM in these biofilms, and the similar iRep distributions between planktonic and biofilm communities suggest that the biofilms contributed substantially to the observed sulfate reduction.

The bioreactor communities comprised largely of Pseudomonadota, Bacillota, Bacteroidota, Synergistota, and Spirochaetota (Figure 7b and Supplementary Data 24). Although these phyla were represented across bioreactor environments, the specific organisms classified to these phyla that made up these communities varied across the different environments. We find generalists, organisms occurring at high mean abundances and present in a large fraction of communities, largely consisted of Bacteroidota and Synergistota (Figure 7c) and lacked lactate dehydrogenases. These organisms were, therefore, likely supported by electron donors common across all six reactor systems, namely, yeast extract, citrate, acetate, and perhaps dead cell biomass. Several Desulfovibrionia SRM could be considered generalists, present across all bioreactors and particularly abundant in planktonic communities. These included Desulfovibrio_desulfuricans_58_43 and Desulfomicrobium_baculatum_60_28 (Figure 7d,e). These SRM are likely to be more metabolically and kinetically versatile, able to consume lactate in the lactate systems and acetate in the lactate and acetate systems, in addition to other available electron donors. The specialist organisms in our bioreactors, those found in a limited number of environments but occur at high abundances in the particular environments, were typically Pseudonomata, capable of oxygen- and nitrate-linked sulfide oxidation as well as fermentation, and Spirochaetota, which are obligate fermenters. Notably, Desulfobacterales_57_55 and Desulfobulbus_propionicus_52_45, both SRM, were predominantly found in high abundance in the biofilms of the acetate reactors, and Desulfovibrio_65_16 was only in high abundance in the lactate well-mixed reactors (stirred tank and channel reactor).

Many Desulfovibrionia were found to be abundant in lactate-supplemented bioreactors and planktonic communities but lower in biofilms and acetate systems (Figure 7e). In contrast, the SRM that were specifically abundant in regions where acetate was available but lactate not was largely isolated to biofilm communities (Figure 4b). Few SRM were found in high abundance in the planktonic communities where acetate was present and lactate absent, indicating that acetate removal by SRM, often at low sulfate concentrations, is likely aided by the formation of biofilms.

### Acetate-Stirred Tank Reactor: A Case Study

The acetate CSTR exhibited a number of physiochemical characteristics that were common across the bioreactors. It is also the simplest of the six systems, supporting only actively dividing planktonic cells in a uniform, mixed environment, with only acetate, yeast extract, and citrate as organic electron donors.

**Figure 8:**
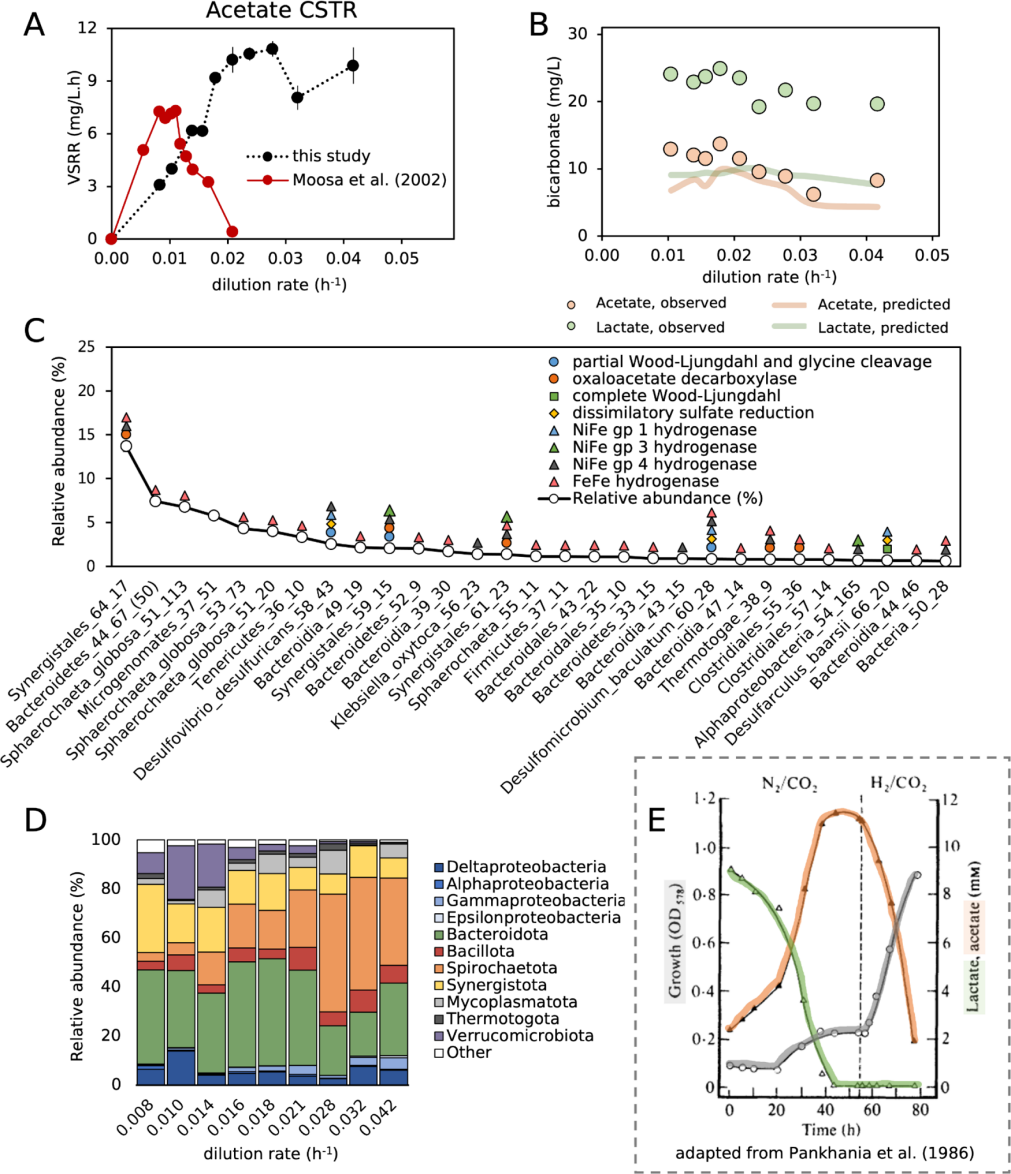
(A) Volumetric sulfate-reducing performance of the acetate-supplemented stirred tank reactors (CSTR) of this study and that of Moosa et al.^20^ (B) The bicarbonate concentrations in acetate- and lactate-supplemented CSTR of this study was measured and independently predicted based on the observed sulfate reduction and quantified lactate fermentation reactions. (C) The widespread distribution of hydrogen-evolving hydrogenases and the potential of the SRM present to take up hydrogen using NiFe group 1 hydrogenases and the ability to fix CO_2_ is evident in the rank abundance curve of the CSTR metagenome at a four-day HRT. (D) The composition of the CSTR community was determined at each steady state using 16S rRNA gene amplicon sequencing and shows the persistent presence of phyla implicated in fermentation from metagenomics in this study. (E) Pankhania et al.^63^ adapted the growth curve of a *D. vulgaris* grown initially on lactate under N_2_/CO_2_ and changed to H_2_/CO_2_ (dotted line), leading to rapid growth and acetate oxidation.

This reactor maintained sulfate reduction at far greater dilution rates (0.042 h^–1^) than had been anticipated based on a previous study^20^ using an acetate-supplemented stirred tank reactor and similar operating conditions but without the inclusion of yeast extract and citrate (Figure 8A). This previous reactor exhibited washout of SRM at a dilution rate of 0.021 h^–1^ (two-day HRT). This change in maximum growth rate of supported SRM represents the impact of yeast extract and citrate on our system. It is very likely that components of the yeast extract directly conferred elevated growth rates to the SRM in our stirred tank reactor. This would represent direct competition between the SRM and fermentative organisms for these compounds. In addition, it is likely that these two groups of bacteria grew syntrophically, whereby the hydrogen produced through fermentation was then consumed by the SRM. This would be beneficial for the fermentative microorganisms as consumption of produced hydrogen maintains low hydrogen partial pressures and allows fermentation to remain energetically favorable.^30,31^

Metagenomic analysis of the acetate-supplemented stirred tank reactor community at the four-day HRT found (Figure 8C) that the most abundant bacteria in the reactor had fermentation-based metabolisms. The community was dominated by a Synergistota (genome Synergistales_64_17) as well as one Bacteroidota and three Spirochaetota. All of the genomes of these abundant bacteria encoded hydrogen-evolving FeFe hydrogenases and encoded a variety of genes relating to amino acid metabolism (Supplementary Data 29). The dominance of these fermentative microorganisms is therefore thought to be afforded through the utilization of yeast extract and possibly citrate, with hydrogen being used as the terminal electron acceptor. We monitored the composition of these communities using 16S rRNA gene amplicon sequencing and found the sustained presence of the fermentative hydrogen-evolving phyla (Figure 6) Bacteroidota, Spirochaetota, and Synergistota at further reduced HRT (Figure 8D).

Three predominant SRM were identified in this stirred tank reactor community at a four-day HRT (Figure 8C), *Desulfomicrobium baculatum*, *Desulfovibrio desulfuricans*, and *Desulfarculus baarsi*. These SRM were widespread in each of the six reactors, with *D. baculatum* and *D. desulfuricans* particularly prevalent in all acetate- and lactate-supplemented planktonic communities. Each of these genomes encoded lactate dehydrogenases, group 1 NiFe hydrogenases, either a full or partial Wood–Ljungdahl pathway with a glycine cleavage pathway,^67,68^ as well as acetyl-CoA synthetases (EC:6.2.1.1), indicating their capacity for acetate metabolism.

The absence of methanogens and the positioning of the SRM in this community, with the nearly ubiquitous distribution of hydrogen-evolving hydrogenases among the non-SRM suggests that these SRM were likely the only organisms consuming hydrogen generated by these fermentative microorganisms while sourcing carbon predominantly from acetate. Simultaneous hydrogen and acetate consumption by SRM has been documented in pure and coculture experiments,^7,63,69^ and hydrogen oxidation linked to sulfate reduction is highly energetically favorable even at low hydrogen concentrations.^70^ In fact, supplementing hydrogen to acetate-oxidizing sulfidogenic cultures has been suggested by Stams and Plugge^66^ to allow better enrichments of SRM. This form of metabolism in SRM typically occurs with assimilation of CO_2_ into biomass. Pankhania et al.^63^ demonstrated that three SRM could be prompted to use acetate and CO_2_ as carbon sources after being supplied with gaseous hydrogen sparged into the reactor headspace (Figure 8E).

We found lower than expected bicarbonate concentrations in our acetate-stirred tank reactor. We measured the concentration of bicarbonate in our acetate- and lactate-supplemented stirred tank reactors at each steady state (Figure 8B). We stoichiometrically predicted the bicarbonate concentration in both reactors based on the observed sulfate reduction linked to acetate and lactate oxidation, respectively (eqs 1 and 3), as well as the degree of fermentative oxidation of lactate (eq 5), which was predicted through the quantification of observed propionate. These estimates are expected to account for all bicarbonate-producing reactions except for citrate and yeast extract oxidation. In the lactate-supplemented reactor, where sulfate reduction could be stoichiometrically linked to lactate oxidation, the observed bicarbonate concentrations were, on average, 9 mg/L higher than predicted. However, in the acetate system, the average difference was only 3 mg/L and sometimes as low as 1.2 mg/L. Consequently, the bicarbonate produced from yeast extract and citrate oxidation seems to be largely unaccounted for in the acetate-stirred tank reactors based on our initial assumption that sulfate reduction was linked to acetate oxidation alone (eq 1). The SRM in this study encoded many mechanisms to assimilate CO_2_ but energetically would not have been able to perform this using acetate oxidation alone.

We posit that the SRM in our acetate-supplemented communities would be using hydrogen generated through fermentation as an electron donor and using acetate and CO_2_ as carbon sources. This is contrary to our initial assumption that SRM were using solely acetate as the electron donor (eq 1). Additionally, although acetate removal is tracked with sulfate reduction, hydrogen and CO_2_ consumption could be taking place at the same time. Hallbeck^64^ noted that the growth rate of their *D. vulgaris* was similar when grown on lactate compared with when supplied with hydrogen, acetate, and CO_2_. In the lactate-supplemented packed bed reactor, this could explain the constant first-order reaction order of sulfate reduction and why the rate of sulfate reduction was not affected by the depletion of lactate. This observation of similar growth rates could in part account for the sustained presence of SRM in the acetate-supplemented stirred tank reactor at elevated dilution rates and account for the depleted bicarbonate concentrations in the reactor.

These results suggest that the VSRR of lactate- and acetate-supplemented BSR reactors could be enhanced through supplementation of low concentrations of fermentable substrates to support a coexisting fermentative, hydrogen-evolving, microbial consortium. If correct, then this approach could be a cost-effective strategy to enhance the growth of the SRM within BSR bioreactors.

## Methodology

### Microbial Culture

The six reactor systems were inoculated with a single mixed sulfidogenic microbial culture described by Hessler et al.^37^ This microbial culture was maintained, prior to inoculation, on neutral modified Postgate B medium (0.42 g/L KH_2_PO_4_, 1.0 g/L NH_4_Cl, 1.0 g/L MgSO_4_.7H_2_O, 0.9 g/L Na_2_SO_4_, 0.4 g/L yeast extract, 0.3 g/L sodium citrate) supplemented with various electron donors including acetate, lactate, ethanol, and algal anaerobic digestate.

### Reactor Configurations and Operation

Three different lab-scale bioreactor configurations were operated in this study, namely, continuous stirred tank reactors, linear flow channel reactors, and up-flow anaerobic packed bed reactors. More detailed information including photographs and diagrams of these bioreactors are shown in the Supporting Information (Figures S1–S5). Each of these reactor systems was operated in duplicate and continuously fed the modified postgate B medium but supplemented with either sodium acetate (0.92 g/L) or sodium lactate (1.2 g/L) as the electron donor. We also included citrate (0.23 g/L) and (0.4 g/L) yeast extract in the media, intended to prevent salt precipitation and as a nutrient source, respectively. The performance of the lactate-stirred tanks and packed bed configuration reactor performance are described by Hessler et al.,^37^^,^^39^ and the acetate channel reactor performance is described by Hessler et al.^38^ The reactors were each inoculated, simultaneously with a mixed microbial sulfidogenic culture sourced from numerous long-term enrichment cultures, and initially operated in batch for a period of 7 days before being operated continuously at a four-day HRT. The methanogenic inhibitor BESA was added to the bioreactors upon inoculation to 10 mM to inhibit methanogenesis. Upon steady-state conditions, defined below, the bioreactors were sampled for solution chemistry, biomass quantification, and genomic DNA. The applied hydraulic retention times were then iteratively reduced following each steady-state condition. This procedure was performed for the HRT: 4, 3, 2.66, 2.33, 2, 1.5, 1.3, and 1.0 days. The measurements taken and the day of sampling post inoculation can be found in the Supporting Information (Supporting Information, data 1–19).

### Analytical Methods

The sulfide produced and the residual sulfate in the reactors were quantified using the DMPD^71^ and APHA (1975) turbidimetric methods,^72^ respectively. The residual and generated volatile fatty acids in the reactor, including acetate, lactate, propionate, citrate, butyrate, and valerate, were quantified by HPLC as described by Hessler et al.^37^ The pH was monitored using a Cyberscan 2500 micro pH meter fitted with an XS Sensor 2-Pore T DHS pH probe, and the redox potential of each sample was measured using the Metrohm 827 pH lab meter fitted with a Pt-ring KCl electrode (Metrohm model 6.0451.100).

### Sulfate Reduction Calculations and Modeling

Sulfate conversion was defined as the sulfate concentration leaving a reactor or zone divided by the sulfate concentration entering the reactor or zone. The sulfate reduction rate was calculated according to eq 7, where *r*_A_ is the sulfate reduction reaction rate (mg·L^–1^. h^–1^), *V* is the volume (L) of the reactor or zone, *X* is equal to the observed sulfate conversion, *F* is the applied flow rate (L·h^–1^), which is equal to the reactor volume divided by the HRT, and *C*_0_ is the concentration of sulfate entering the reactor or zone (mg/L)
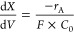
7

The sulfate reduction rates exhibited by each packed bed reactor were modeled as irreversible *n*^th^-order and first-order reactions^39^ (eq 8) along an ideal plug-flow reactor according to derived eqs 9 and 10, respectively, where *r*_A_ is the sulfate reduction reaction rate, *V* is the volume of the reactor or zone, *F* is the applied flow rate, *C*_0_ is the concentration of sulfate entering the reactor or zone, *n* is the reaction order, and *k* is the rate constant. Nonlinear regression using the iterative, generalized reduced gradient method, employed by SOLVER (Microsoft Excel, Microsoft Office 365 ProPlus), was used to solve for the rate constant and reaction order. Further details are discussed in Hessler et al.^39^
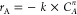
8
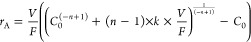
9
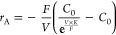
10

### Steady-State Biological Sampling

The steady state was defined when the residual sulfate concentration leaving a reactor and each of its zones was consistent over a minimum period of three HRT. Biofilm and planktonic cells were isolated for metagenomic sequencing and cell quantification from the reactors at a steady state at a four-day HRT. All cell sampling steps were performed in duplicate. Solid support structures were removed from the LFCR and UAPBR using sterile stainless-steel scissors and forceps. The biofilm-associated cells were recovered through gentle agitation in a fresh sterile reactor medium. Cells embedded in the biofilms attached to the solid support structures were isolated in duplicate for cell quantification using a nondestructive whole cell detachment protocol using the nonionic detergent tween20 and vigorous agitation as described by Hessler et al.^37^ The cells from each of these defined phases were quantified, in duplicate, through direct cell counting oil immersion phase contrast microscopy (Olympus BX40) and a Thoma counting chamber. Recovered planktonic and biofilm-associated cells were recovered for total DNA extraction by centrifugation at 10,000*g* for 10 min. Total DNA extraction of biofilm attached cells was performed directly from the colonized solid support structures.

### Metagenomic DNA Extraction and Sequencing

Total genomic DNA was extracted from 34 metagenomic samples isolated at a four-day HRT, representing the 16 bioreactor samples from the six bioreactors and the inoculum sample, in duplicate, using a NucleoSpin Soil Genomic DNA extraction kit (Machery-Nagel, Germany) as per manufacturer’s instructions. Library preparation and Illumina sequencing were performed at UC Berkeley’s Functional Genomics Laboratory. Paired-end Illumina sequence libraries were prepared with an insert size of 400–800 bp and were sequenced on an Illumina HiSeq4000 generating 2.5 Gbp of raw data per sample.

### Metagenomic Read Processing, Assembly, and Annotation

All read sets were trimmed using Sickle^73^ (2011, https://github.com/najoshi/sickle) using default parameters. Sequencing reads originating from metagenomes sampled in duplicate were concatenated into single files and were processed in parallel with the two original unconcatenated read sets. Reads were assembled using MEGAHIT^74^ version 1.1.3 with the following parameters: --k-min 21, --k-max 99, --k-step 10, --min—count 2. Read mappings for all scaffolds were determined using Bowtie2.^75^ Open reading frames (ORFs) were predicted using Prodigal’s metagenome procedure.^76^ These ORFs were then annotated using USEARCH^77^ (Edgar, 2010) against KEGG^78^ (Kanehisa and Goto, 2000), UniRef100, and Uniprot (Consortium, 2009) databases. Functional genes were predicted using Hmmsearch (http://hmmer.org/) against in house Hidden Markov Models (HMM, https://github.com/banfieldlab/metabolic-hmms) built from KEGG orthology groups, TIGRFAM, and PFAM databases. Metabolic genes studied in the current study were similar to those used by METABOLIC^79^ software package.

### 16S rRNA Gene Amplicon Sequencing

The composition of the microbial communities of the six bioreactors were assessed using 16S rRNA gene sequencing at each tested HRT. The V3–V4 region of the 16S rRNA gene was amplified from extracted genomic DNA by polymerase chain reaction using primers FwOvAd_341F (5̀-TCGTCGGCAGCGTCAGATGTGTATAAGAGACAGCCTACGGGNGGCWGCAG-3̀) and ReOvAd_785R (5̀-GTCTCGTGGGCTCGGAGATGTGTATAAGAGACAGGACTACHVGGGTATCTAATCC-3̀) and the following conditions: 95 °C for 3 min; 25 cycles of denaturation at 95 °C for 30 s, annealing at 55 °C for 30 s, elongation at 72 °C for 30 s; and a final extension at 72 °C for 4 min. Amplicon libraries were then sequenced on an Illumina MiSeq to yield 300 bp paired-end reads. The processing of Illumina reads, the picking of operational taxonomic units (OTUs), and taxonomic classification of the OTUs were performed as described by Hessler et al.^37^ The 16S rRNA gene sequences are available at GenBank under the accession numbers MH603613–MH603682. The relative abundance data can be found in the Supporting Information, data 30.

### Hydrogenase Classification and Phylogeny

The protein sequences of the catalytic FeFe and NiFe hydrogenase subunits were identified using HMMs described above and were aligned with Muscle^77^ v3.8.31 (Edgar, 2004) and trimmed in Geneious v2019.0.4. Maximum-likelihood phylogenetic trees for NiFe and FeFe hydrogenase protein sequences were constructed using Fasttree2.^81^ The hydrogenases were classified based on the best-hit HMM and the presence of consensus motifs surrounding the conserved cysteine residues and further verified using HydDB.^80^ Protein sequences classified as group A FeFe hydrogenases were further classified to group A1, A2, A3, or A4 based on the downstream catalytic subunit protein sequence.^82^

### Binning of metagenomes

Individual binning was performed on each of the 17 concatenated and 34 unconcatanated assemblies using MaxBin,^82^ Metabat,^83^ and Concoct^84^ and manual binning performed on the basis of GC content, coverage and consensus taxonomy using ggKbase (ggkbase.berkeley.edu). Series coverage information across the 51 samples was used for binning in parallel using Concoct. A set of nonredundant genome bins were generated for each metagenome using Das Tool^85^ and a nonredundant set of genome bins across the 51 assemblies was selected using dRep.^86^ The degree of genome completeness and contamination was determined using CheckM^87^ based on the recovery of bacterial and archaeal single copy genes. This was repeated using CheckM’s CPR specific gene marker set for the evaluation of a recovered Microgenomates genome bin.

### Relative Abundance

The relative abundance of each organism per sample was determined using CoverM (coverm genome -m relative abundance, https://github.com/wwood/CoverM), which maps reads to binned contigs and determines the total fraction of reads mapping to each genome normalized by genome size. This value is subsequently multiplied by the proportion of the total reads that were mapped to a genome.

### Indices of Replication

Relative instantaneous microbial growth rates were estimated using the iRep. These were calculated using iRep.py,^65^ using default settings, for all genomes that passed iRep’s quality threshold.

### Phylogenetic analyses

Phylogenetic analyses were performed by using 16 universal ribosomal proteins. Sixteen ribosomal protein sequences from 2896 reference organisms, as described by Hug et al.,^88^ and each of the recovered genome bins were aligned using MUSCLE^77^ v3.8.31, trimmed using Geneious v2019.0.4., and subsequently concatenated into a single alignment. A maximum-likelihood phylogenetic reconstruction of these two alignments were performed using IQtree^89^ (-st AA -nt 48 -bb 1000 -m LG + G4 + FO + I).

## Data Availability

The genomes and raw sequencing are available under the NCBI project number PRJNA728813 (See supplementary data 22 for genome accession numbers). Genomes will also be made available at https://ggkbase.berkeley.edu/Biological_sulfate_reduction_analysis/organisms.

## Supporting Information Available

A detailed description of the bioreactors used in this study together with additional schematics and photographs (PDF)
CTSR performance data, LFCR performance data, UAPBR performance data, metagenomics data, and 16S rRNA gene amplicon sequencing data (XLSX)
Permission request form for use of figures/tables from publishers other than ACS (PDF)
