# Supplementary material for: Harnessing Fermentation May Enhance the Performance of Biological Sulfate-Reducing Bioreactors
Source: Environ Sci Technol. 2024 Feb 1;58(6):2830–46. doi: 10.1021/acs.est.3c04187 (PMC10867827; doi:10.1021/acs.est.3c04187)
Supplement: Supplementary file 1 — es3c04187_si_001.pdf [file es3c04187_si_001.pdf]

Supplementary information summary

Harnessing fermentation may enhance the performance of biological sulfate-reducing bioreactors

Tomas Hessler[1,2,3,4], Susan T.L. Harrison[1,5*], Jillian F. Banfield[2,3,6,*], Robert J. Huddy[1,5*]

[1]The Center for Bioprocess Engineering Research, University of Cape Town, South Africa
[2]The Innovative Genomics Institute at the University of California, Berkeley, California, USA
[3]The Department of Earth and Planetary Science, University of California, Berkeley, California, USA
[4]Environmental Genomics and Systems Biology Division, Lawrence Berkeley National Laboratory, Berkeley, CA, USA
[5]The Future Water Institute, University of Cape Town, South Africa
[6]The Department of Environmental Science, Policy and Management, University of California, Berkeley, California, USA
[*] corresponding author

30 Supplementary Tables
5 Supplementary Figures

Contents

CSTR performance data

Supplementary data 1. The sulfate conversion (Xs) of the feed 1000 mg/L sulfate and the achieved VSRR achieved by the acetate and lactate CSTRs at steady state at each applied HRT.

Supplementary data 2. The residual sulfate and produced sulfide concentrations recorded at steady state at each HRT.

Supplementary data 3. The volatile fatty acid concentrations were determined by HPLC from the acetate and lactate-supplemented continuous stirred tank reactors at steady state at each applied HRT. Citrate was never detected in these bioreactors at any HRT.

Supplementary data 4. The mean pH and redox potential measurements from the acetate and lactate supplemented continuous stirred tank reactors recorded at steady state at each applied HRT

Supplementary data 5. The volatile fatty acid concentrations determined by HPLC from the acetate and lactate supplemented continuous stirred tank reactors during steady state at each applied HRT. Citrate was never detected in these bioreactors at any HRT.

Supplementary data 6. Planktonic cell counts from the acetate- and lactate-supplemented stirred tank reactors at steady state at each applied HRT.

LFCR performance data

Supplementary data 7. The observed sulfate and sulfide concentrations together with the stoichiometrically predicted sulfide concentration in the acetate- and lactate-supplemented LFCR at steady state at each applied HRT.

Supplementary data 8. The residual sulfate and produced sulfide concentrations recorded at steady state at each HRT from the acetate and lactate-supplemented LFCRs.

Supplementary data 9. The mean pH and redox potential measurements from the acetate and lactate-supplemented LFCR reactor zones were recorded at steady state at each applied HRT.

Supplementary data 10. the mean volatile fatty acid concentrations determined by HPLC from the acetate and lactate-supplemented LFCRs at steady state at each applied HRT. Citrate was never detected in these bioreactors at any HRT.

Supplementary data 11. The mean planktonic cell density in the acetate and lactate LFCR reactor zones at steady state at each applied HRT

Supplementary data 12. The attached and associated cell density associated with the biomass support structures of the acetate and lactate-supplemented LFCRs at steady state at a one- and four-day HRT. Biofilms were not sampled between these HRT to limit disruption to reactor performance from oxygen ingress.

UAPBR performance data

Supplementary data 13. The residual sulfate and produced sulfide concentrations recorded at steady state at each HRT for the acetate and lactate-supplemented UAPBRs.

Supplementary data 14. The observed sulfate and sulfide concentrations together with the stoichiometrically predicted sulfide concentration in the acetate and lactate-supplemented UAPBR at steady state at each applied HRT.

Supplementary data 15. The mean pH of the acetate and lactate supplemented UAPBR reactor zones recorded at steady state at each applied HRT.

Supplementary data 16. The mean redox potential measurements from the acetate and lactate supplemented UAPBR reactor zones recorded at steady state at each applied HRT

Supplementary data 17. The mean volatile fatty acid concentrations determined by HPLC from the acetate and lactate supplemented UAPBRs at steady state at each applied HRT. Citrate was never detected in these bioreactors at any HRT.

Supplementary data 18. The attached and associated cell density associated with the biomass support structures of the acetate and lactate-supplemented UAPBRs from each subzone at steady state at a one-day HRT.

Supplementary data 19. The attached and associated cell density associated with the biomass support structures of the acetate and lactate-supplemented UAPBRs from each subzone at steady state at a four-day HRT.

Metagenomics

Supplementary data 20. Genome statistics and the taxonomy associated with each recovered metagenome-associated genome recovered from the BSR bioreactor metagenomes.

Supplementary data 21. Assembly statistics associated with the metagenomes sampled from the six BSR bioreactors at a four-day HRT steady state. Approximately 5 Gbp of sequencing reads were generated for each metagenome.

Supplementary data 22. NCBI accession number associated with each MAG recovered from the BSR bioreactors of this study.

Supplementary data 23. The classification of the 162 microbial genomes recovered in this study, as determined by GTDB-tk

Supplementary data 24. The relative abundance (%) of the 162 microbial genomes across the six bioreactors at a 4 day HRT steady state as well as the original inoculum.

Supplementary data 25. Presence and absence table showing major metabolic genes encoded by the 162 microbial genomes of this study, as determined using HMMs described in the Methodology.

Supplementary data 26. Presence and absence of metabolic genes encoded by the 162 microbial genomes of this study, as represented in Figure 4.

Supplementary data 27. Presence and absence of specific NiFe and FeFe hydrogenase genes encoded by the 162 microbial genomes of this study.

Supplementary data 28. Presence and absence of metabolic genes encoded by the 22 SRM genomes of this study, as represented in Figure 5.

Supplementary data 29. Presence and absence of amino acid metabolism genes encoded by the recovered genomes of this study.

16S amplicon sequencing

Supplementary data 30. 16S rRNA gene amplicon sequence relative abundance data associated with the communities of the six bioreactors of the BSR reactors at steady state at several applied HRT.

Bioreactor configurations

Figure S1. Schematic diagram of the Continuous stirred-tank reactor operated in this study. A: feed reservoir; B: pump; C: feed inlet; D: sampling port; E: effluent port; F: effluent collection; G: glass heating jacket; H: Rushton impellers; I: CSTR

Figure S2. Schematic diagram of the Linear flow channel reactor (LFCR) operated in this study. The reactor had a working volume of 2.4ℓ and a headspace of 1.25ℓ. A: feed reservoir; B: pump; C: feed inlet; D: effluent port; E: effluent collection; G: sampling port; H: internal heating coil; I: carbon fibre support scaffold; J: carbon microfibres which extend across the reactor, anchored to the carbon fibre support scaffold, K: inlet zone; L: effluent zone.

Figure S3. Photographs of the Perspex LFCR (A) during assembly and (B) after carbon microfibres had been fitted within the reactor, prior to inoculation.

Figure S4. Schematic diagram of the 1ℓup-flow anaerobic packed bed reactors (UAPBRs) operated in this study. The reactor had a working volume of 1ℓ, an internal diameter of 40mm and a height of 80 mm. The reactor was demarcated into three 0.33ℓ sequential zones namely the inlet, middle and effluent zone, and was also demarcated into six 0.167 ℓ subzones number from one at the inlet to six at the effluent. A: feed reservoir; B: pump; C: feed inlet; D: sampling port; E: effluent port; F: effluent collection; G: glass heating jacket; J: inlet zone; K: middle zone; L: effluent zone.

Figure S5. Photographs of the acetate- (right) and lactate-supplemented (left) UAPBR experimental set up of this study during continuous operation.

Continuous stirred-tank reactor

This study used two identical glass stirred-tank reactors with a working volume of 1ℓ (Figure 1) The reactors had a height of 200 mm, a diameter of 104 mm and a liquid height of 118 mm. Mixing was achieved using a Heidolph overhead stirrer powered a four-blade Rushton impeller (D/T = 0.303) and was operated at 300 rpm. Vortex formation was prevented using four vertical baffles. The lids of the reactors were modified to prevent air ingress into the reactors: the shaft of the impellor passed through a column in the lid and was held in place by three rubber O-rings. The reactors were operated continuously by means of a variable speed peristaltic pump which pumped feed solution through the inlet port which extended to near the base of the reactor. Sampling was performed using a single sampling port which extended to near the base of the reactor. Effluent was discharged from the reactor via the effluent port by gravity. The reactors were maintained at 30°C using water passed through external glass jackets, by a circulating water bath.

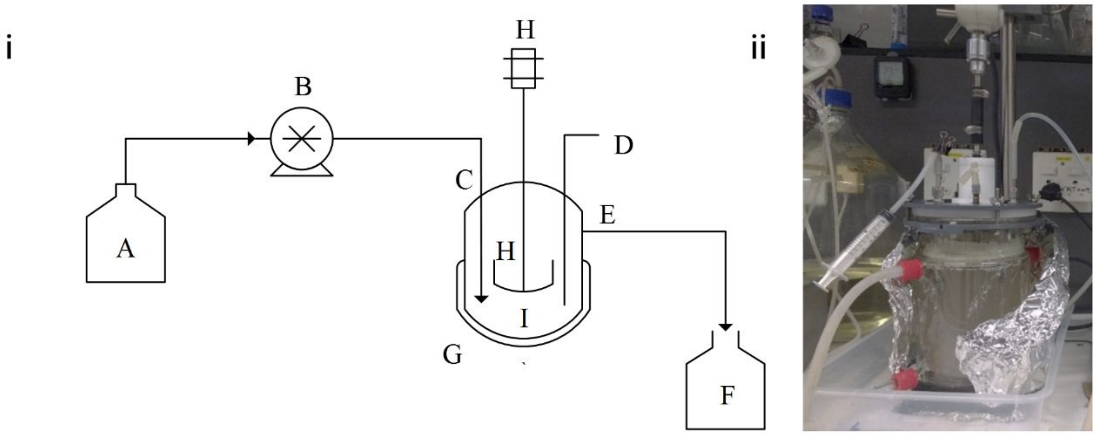

Figure S1 Schematic diagram of the Continuous stirred-tank reactor operated in this study. A: feed reservoir; B: pump; C: feed inlet; D: sampling port; E: effluent port; F: effluent collection; G: glass heating jacket; H: Rushton impellers; I: CSTR

Linear flow channel reactor

The 2.4 ℓ Linear flow channel reactors (LFCRs) used in this study (Figure 2, Figure 3) were constructed from 12 mm thick Perspex. The reactor had external dimensions of length: 275 mm, width: 123 mm and height of 112 mm. The reactor had an internal length of 247 mm, width of 100 mm and height, from base to inlet and effluent ports, of 100 mm. The reactor had a working volume of 2.4 ℓ and a headspace of 1.25ℓ. A silicone gasket was placed between the top of the reactor and the Perspex lid which were securely bolted to the reactors to prevent the ingress of oxygen and prevent the loss of volatile sulphide from the reactor. Heated water was pumped through internal stainless-steel heating coils using a circulating water bath to maintain the temperature within the reactor at 30°C. Carbon microfibres were incorporated into the LFCR to increase the internal surface area for the formation of biofilms within the reactor. These carbon microfibres were anchored to- and extended above and below three stainless-

steel metal frames which spanned the length of the reactor from the inlet to the effluent port as shown in Figure 2 and Figure 3.

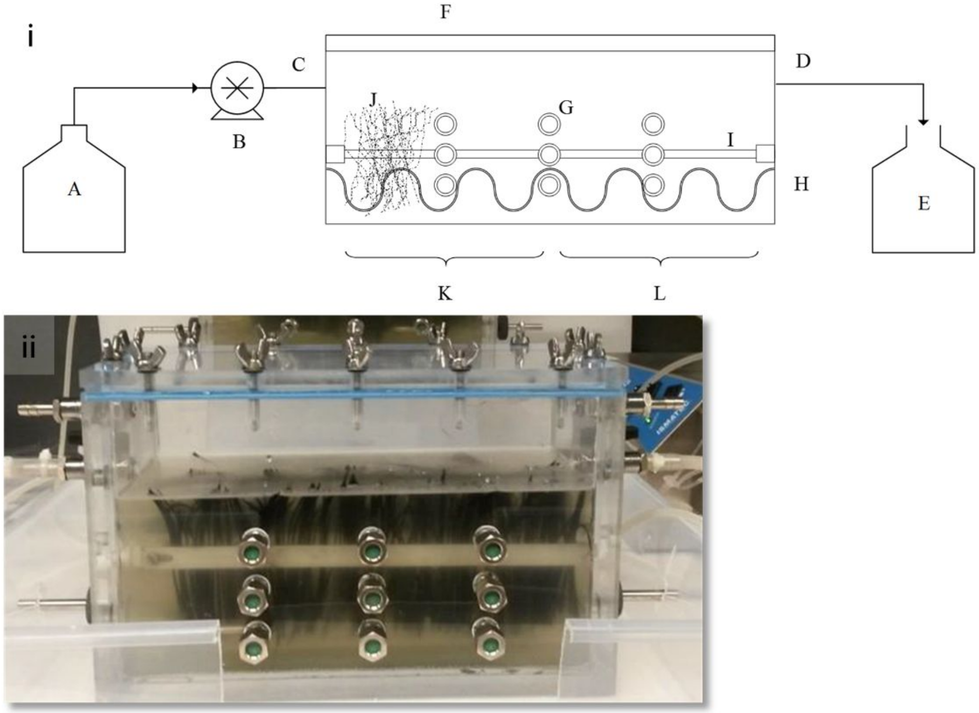

Figure S2 Schematic diagram of the Linear flow channel reactor (LFCR) operated in this study. The reactor had a working volume of 2.4ℓ and a headspace of 1.25ℓ. A: feed reservoir; B: pump; C: feed inlet; D: effluent port; E: effluent collection; G: sampling port; H: internal heating coil; I: carbon fibre support scaffold; J: carbon microfibres which extend across the reactor, anchored to the carbon fibre support scaffold, K: inlet zone; L: effluent zone

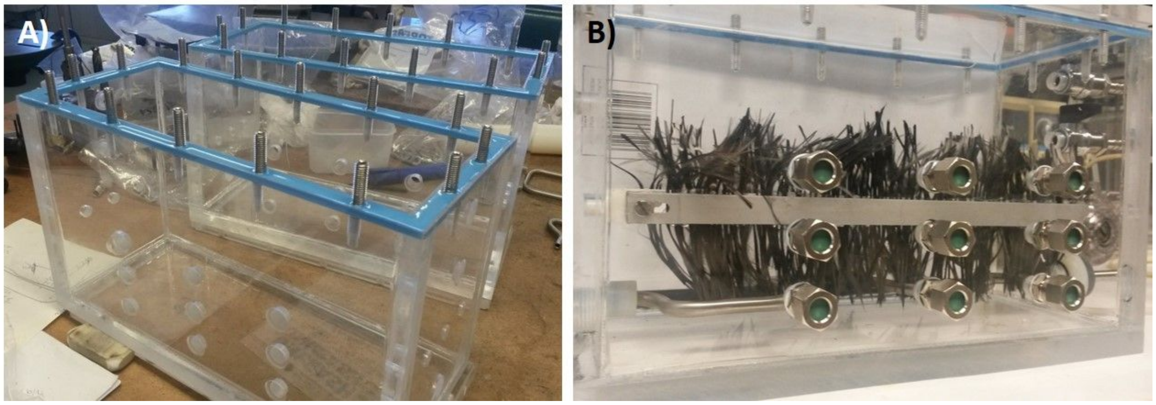

Figure S3 Photographs of the Perspex LFCR (A) during assembly and (B) after carbon microfibres had been fitted within the reactor, prior to inoculation.

Up-flow anaerobic packed bed reactor

Two glass Up-flow anaerobic packed bed reactor (UAPBRs) were operated in this study as shown in Figure 4 and Figure 5. These reactors were held vertical and bolted to a metal frame. Reactor medium was continuously pumped into the reactors using a variable speed peristaltic pump via the inlet port positioned of the base of the reactors. Effluent from these reactors was discharged by gravity via effluent ports positioned near the top of the reactor. Heated water was pumped through external heating jackets using a circulating water bath to maintain the temperature within the reactors at 30°C. Sampling pipes were fitted to the base and top of the reactor which extend to the boundary between the inlet and middle zones, middle and effluent zones and just below the effluent port. These reactors were packed with open-cell polyurethane foam cubes (20 mm x 20 mm) to act as a support matrix for the formation of microbial biofilms within the reactors. This foam occupied 40 cm^3 (4%) of the internal 1ℓ working volume.

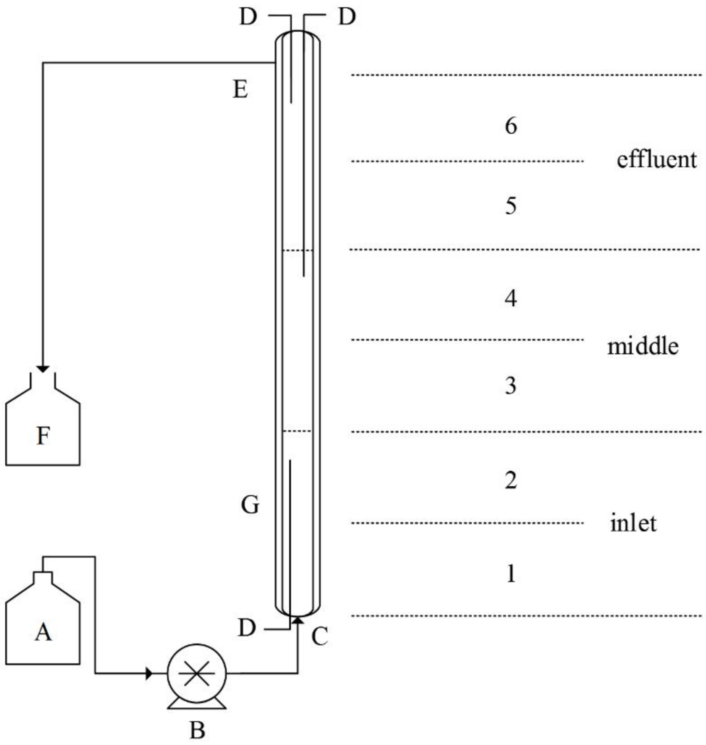

Figure S4 Schematic diagram of the 1ℓup-flow anaerobic packed bed reactors (UAPBRs) operated in this study. The reactor had a working volume of 1ℓ, an internal diameter of 40mm and a height of 80 mm. The reactor was demarcated into three 0.33ℓ sequential zones namely the inlet, middle and effluent zone, and was also demarcated into six 0.167 ℓ subzones number from one at the inlet to six at the effluent. A: feed reservoir; B: pump; C: feed inlet; D: sampling port; E: effluent port; F: effluent collection; G: glass heating jacket; J: inlet zone; K: middle zone; L: effluent zone.

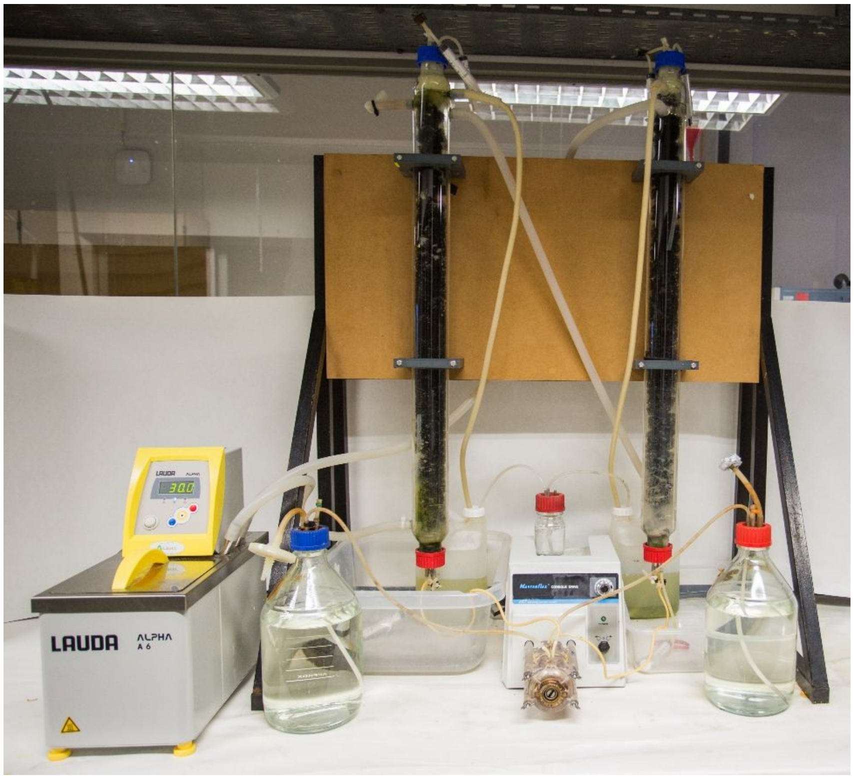

Figure S5 Photographs of the acetate- (right) and lactate-supplemented (left) UAPBR experimental set up of this study during continuous operation.
