# Supplementary material for: Harnessing Fermentation May Enhance the Performance of Biological Sulfate-Reducing Bioreactors
Source: Environ Sci Technol. 2024 Feb 1;58(6):2830–46. doi: 10.1021/acs.est.3c04187 (PMC10867827; doi:10.1021/acs.est.3c04187)
Supplement: Supplementary file 3 — es3c04187_si_003.pdf [file es3c04187_si_003.pdf]

Permission Request Form for Use of Figures/Tables from Publishers Other Than ACS

PERMISSION REQUEST FORM "C"
Request to Use Figures/Tables Published in Non-ACS Publications Only

Date: 13 December 2023

To: bioengineering.editorial.office@frontiersin.org
Publisher's Contact Information
Frontiers
Av. du Tribunal-Fédéral 34
1005 Lausanne

____
Publisher's Fax Number

From: Tomas Hessler
2151 Berkeley Way Berkeley
CA94720 Berkeley
United States
(+1)5103656591

4th January 2024

____
Your Specific Deadline Date

I am preparing a paper entitled:

Harnessing fermentation may enhance the performance of biological sulfate-reducing bioreactors

to appear in

Environmental Science and Technology

____
Title of ACS Publication

which is published by the American Chemical Society, a not-for-profit membership society and a STM signatory.

I would appreciate your permission to use the following material in all formats including but not limited to print, microform, electronic, and/or CD-ROM from the following reference:

From a journal or magazine:

Publication Title | Year | Vol. | No. | Page(s) | Material to be used

Integrated Kinetic Modelling and Microbial Profiling Provide Insights Into Biological Sulfate-Reducing Reactor Design and Operation.(2022). *10*, 897094. Figure 5. (Frontiers in Bioengineering and Biotechnology)

____

From a book: include book title, series name and number, year, page(s), book editor(s) name(s), chapter author's name(s), and material to be used, such as Figs. 2 & 3, full text, etc.

____
____

If you have a required **credit line,** please give it below:

____

For your convenience, you may sign and date the bottom of this letter to indicate your agreement. Please return this form to me at the above fax number or address, by the above **deadline date** (if given above). Thank you.

Agreed: Dr Michiel B. Dijkstra (ScienceComms specialist) **Date:** 22 Dec 2023

Publisher's Signature

Form Revision #06272008
